# Optical Anisotropy in van der Waals materials: Impact on Direct Excitation of Plasmons and Photons by Quantum Tunneling

**DOI:** 10.1038/s41377-021-00659-7

**Published:** 2021-11-08

**Authors:** Zhe Wang, Vijith Kalathingal, Thanh Xuan Hoang, Hong-Son Chu, Christian A. Nijhuis

**Affiliations:** 1 Department of Chemistry, National University of Singapore, 3 Science Drive 3, Singapore, 117543 Singapore; 2 Centre for Advanced 2D Materials, National University of Singapore, 6 Science Drive 2, Singapore, 117564 Singapore; 3 Department of Electrical and Computer Engineering, National University of Singapore, 4 Engineering Drive 3, Singapore, 117583 Singapore; 4 Department of Electronics and Photonics, Institute of High Performance Computing, A*STAR (Agency for Science, Technology and Research), 1 Fusionopolis Way, Singapore, 138632 Singapore; 5 Hybrid Materials for Opto-Electronics Group, Department of Molecules and Materials, MESA+ Institute for Nanotechnology and Center for Brain-Inspired Nano Systems, Faculty of Science and Technology, University of Twente, 7500 AE Enschede, The Netherlands

**Keywords:** Nanophotonics and plasmonics, Sub-wavelength optics

## Abstract

Inelastic quantum mechanical tunneling of electrons across plasmonic tunnel junctions can lead to surface plasmon polariton (SPP) and photon emission. So far, the optical properties of such junctions have been controlled by changing the shape, or the type of the material, of the electrodes, primarily with the aim to improve SPP or photon emission efficiencies. Here we show that by tuning the tunneling barrier itself, the efficiency of the inelastic tunneling rates can be improved by a factor of 3. We exploit the anisotropic nature of hexagonal boron nitride (hBN) as the tunneling barrier material in Au//hBN//graphene tunnel junctions where the Au electrode also serves as a plasmonic strip waveguide. As this junction constitutes an optically transparent hBN–graphene heterostructure on a glass substrate, it forms an open plasmonic system where the SPPs are directly coupled to the dedicated strip waveguide and photons outcouple to the far field. We experimentally and analytically show that the photon emission rate per tunneling electron is significantly improved (~ ×3) in Au//hBN//graphene tunnel junction due to the enhancement in the local density of optical states (LDOS) arising from the hBN anisotropy. With the dedicated strip waveguide, SPP outcoupling efficiency is quantified and is found to be ∼ 80% stronger than the radiative outcoupling in Au//hBN//graphene due to the high LDOS of the SPP decay channel associated with the inelastic tunneling. The new insights elucidated here deepen our understanding of plasmonic tunnel junctions beyond the isotropic models with enhanced LDOS.

## Abstract (web summary)

Enhancement in local density of optical states arising from the optical anisotropy of hexagonal boron nitride significantly improves the emission rates of plasmons and photons in quantum mechanical tunnel junctions.

## Graphical abstract

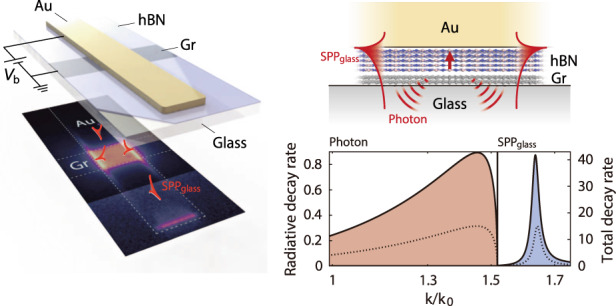

## Introduction

The research field of plasmonics, where intriguing light-matter interaction at the surfaces of metals, doped semiconductors, and two-dimensional (2D) materials, is associated with the hybridized electromagnetic waves and charge density oscillations, called surface plasmon polaritons (SPPs), has attracted significant interest in the past decade^1,2^. Light-matter interaction in both classical, as well as the quantum realm, is involved in plasmon excitation, which is pertinent in the design and optimization of the devices for numerous applications involving nanoscale photoemission^3,4^ such as optoelectronics and photovoltaics^5,6^, single molecule^7^ and biosensing^8^, nanolasing^9,10^, and quantum information processing^11,12^. Mostly, SPPs are excited by optical means, but metal-insulator-metal (MIM) tunnel junctions (TJs) offer an electrical route for the localized and propagating SPP^13–18^ excitation via inelastic electron tunneling (IET) which qualify as an ultrafast non-diffraction limited source of SPPs as well as photons. In plasmonic MIM-TJs, IET mediated SPP or photon emission has been extensively investigated in the past^13,14,16,17,19^ and is the result of an indirect process where tunneling electrons inelastically couple to SPP modes within the highly confined MIM gap (since the insulator has a thickness of just a few nm), the so-called MIM-SPPs^13^, which subsequently decay to single interface SPPs or photons, provided the large momentum-mismatch (Δ*k* *~* 10) is compensated^14,16,20–24^. However, the strong field confinement associated with the MIM-SPPs is accompanied by significant ohmic losses impose a severe lack of control over the design degrees of freedom available for these systems. In contrast to the narrow-band emission from typical quantum emitters, IET in MIM-TJs results in a characteristic broadband SPP and/or photon emission. This non-resonant energy transfer to the metal electrodes or the surroundings^15,25,26^ is analogous to the Purcell effect^27^ for emitters in a plasmonic environment, approximated as a dipole emitter located in the TJ^15^. Therefore, the efficiency of IET mediated SPP or photon outcoupling becomes highly sensitive to the local density of optical states (LDOS)^20,28–30^ and controlling the LDOS remains a critical challenge in the field, especially when the source-spectrum is broadband. The methods reported so far, aiming to achieve the control, have been largely based on changing the shape, or the type of the material of the electrodes^17,18^ all of which are resonant in nature with a narrow-band response and demand a significant control over the device fabrication processes. In this manuscript, by tuning the tunneling barrier itself, we show that the efficiency of the inelastic tunneling rates can be improved by a factor of 3, with concomitant enhancement in SPP and photon emission, by exploiting the effect of the optical anisotropy in plasmonic TJs. Related to the conventional isotropic models, where investigations are focused either on the role of the MIM-SPP modes^15,18^ or on the local TJ geometry^29,31^, our study provides new insights towards controlling the LDOS and enhancement of the outcoupling in IET mediated plasmon emission.

Combining the plasmonic TJs with 2D materials is of particular interest for various reasons^32–36^. In particular, the small thickness of just a few atoms along with the in-plane crystalline and out-of-plane layered nature, makes 2D materials interact efficiently with their plasmonic environments, especially when the field is evanescently confined over subwavelength volumes^37,38^. Of potential interest is hexagonal boron nitride (hBN), a wide bandgap insulator (∼ 5.5 eV)^37,39^ where different in-plane and out-of-plane interactions in the layers lead to a strong optical anisotropy in the optically relevant energy range of 1−2.5 eV^40–42^ and hyperbolicity in the far infra-red^43^. For these reasons, hBN is a potentially interesting tunnel barrier material for plasmonic TJs and a theoretical study towards the effect of the anisotropy on IET in hBN based TJs is of particular interest.

This paper describes the effect of the inherent optical anisotropy of hBN^41^ on the excitation of SPPs and photons from IET^33^ across the hBN layer in Au//hBN//Gr junctions, where Gr (graphene) serves as a transparent electrode, the tunnel barrier is defined by the hBN layer^44^, and “//” indicates a van der Waals interface. The Au counter electrode also functions as an integrated plasmonic strip waveguide for the SPPs. Theoretically, we treat IET across hBN analogous to a quantum emitter embedded in an anisotropic medium^45–47^ where the LDOS is strongly affected by the local dielectric environment^20,28–30^. Importantly, in contrast to the typical MIM-TJ configurations, replacement of one of the metal electrodes with an optically transparent Gr electrode makes the Au//hBN//Gr junction an open plasmonic system where the IET excitation of the photonic and SPP continuum is direct, without an intermediate MIM-SPP mode excitation^33–35^. Therefore, the momentum mismatch in the electron-to-plasmon or photon energy transfer in IET is negligible in these TJs, and the associated field confinement losses will be at a minimum. Compared to the previous study from Parzefall et al.^33^, with a dedicated SPP waveguide directly integrated with the TJ, we quantify the strength of the IET coupling to the SPP continuum. Characterizing the SPP outcoupling pathway is particularly important, considering the potential of the 2D material-metal hybrid systems in on-chip integration and SPP-mediated information processing. We use direct and back focal plane (BFP) imaging to characterize the SPPs, which are corroborated by numerical simulations. Using analytical calculations, we show that the hBN-anisotropy strongly affects the radiative and nonradiative decay rate associated with the IET, leading to an enhanced SPP and photon excitation related to the isotropic case. Experimentally, we observe uniform photon emission from the TJ and SPP outcoupling along the Au strip waveguide, corroborating the direct IET energy transfer process. By comparing the light scattering from the end of the SPP waveguide with the radiation from the TJ area, relative SPP outcoupling is estimated to be ∼ 80% stronger than the radiative outcoupling. The photon emission rate from the Au//hBN//Gr junction shows good qualitative agreement with the anisotropic-hBN model calculations and the numerical simulations. Our findings provide new insights into the mechanisms of SPP and photon excitation and outcoupling in plasmonic tunnel junctions.

## Results

**Fig. 1:**
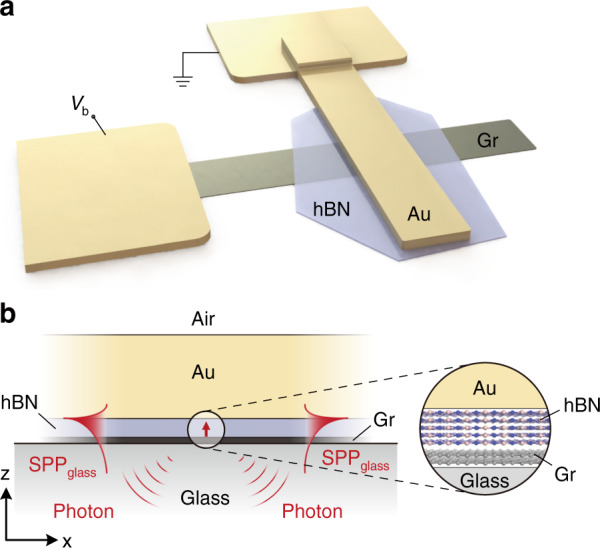
Device schematic. **a** Schematic illustration of the biased Au//hBN//Gr tunneling junction. **b** Cross-section of the junction area with a red-upward arrow representing the dipole. Direct excitation of the SPPs (SPP_glass_) and photons are schematically shown. The zoom-in area illustrates the layered nature of the hBN and few-layer Gr

Fig. 1a shows the schematic illustration of the Au//hBN//Gr junction fabricated on a glass substrate, and we characterized SPP and photon excitation by analyzing the far-field light emission recorded in the real- and Fourier-plane of the leakage radiation. Importantly, in addition to the direct photon emission from the TJ, the integrated Au plasmonic strip waveguide (Fig. 1a) allows us to explicitly investigate the direct SPP excitation, in contrast to the recent studies from Parzefall *et al*. with similar TJs^33^. First, we theoretically investigate the LDOS associated with the IET in the weak coupling limit^15^. Non-resonant energy transfer accompanying the IET is modeled with a two-level dipole emitter, and the energy decay rate is numerically calculated using the system’s dyadic Green’s function^48^.

The dielectric function of the TJ (*ϵ*_TJ_) consists of five regions (Fig. 1b), represented as $\tt \epsilon_{TJ} \,=\, \epsilon _0 - \epsilon _{Au}\left( \omega \right)-\tilde \epsilon _{hBN} - \epsilon _{Gr} - \epsilon _d$, where *ϵ*_0_ = 1 (air) and $\tt \epsilon _{Au}\left( \omega \right) =\, \epsilon _{Au}^\prime \left( \omega \right) + {\it i}\epsilon _{Au}^{\prime\prime} \left( \omega \right)$ represents the complex dielectric function of Au^49^. The uniaxial anisotropic dielectric function of the hBN layer is represented as^50^ (See Supplementary Section 1)

1 $$\tt \tilde \epsilon _{hBN} = \left( {\begin{array}{*{20}{c}} { \epsilon _{||}} & 0 & 0 \\ 0 & { \epsilon _{||}} & 0 \\ 0 & 0 & { \epsilon _ \bot } \end{array}} \right)$$

where $\epsilon_{||}$ and *ϵ*_⊥_ are both real and positive^40^, $\tt \epsilon _{Gr} \,= \,\epsilon _{{{Gr}}}^\prime \left( \omega \right) + {\it i} \epsilon_{Gr}^{\prime \prime }\left( \omega \right)$ represents the complex dielectric function of graphene^51^, and *ϵ*_*d*_ = 2.31 (glass). In the time-harmonic limit (e^−*iωt*^), the current density ***J*** associated with IET can be represented as^48^ ***J***(***r***) = −*iω****μ****δ*(***r***−***r***_0_), where ***μ*** represents the dipole moment for a spatially localized dipole at the center of the hBN layer at ***r*** = ***r***_**0**_ (red vertical arrow in Fig. 1b) and *δ*(***r***−***r***_**0**_) represents the Dirac delta function. From the source terms ***J*** and ***μ***, the rate of energy loss (*dW/dt*) associated with the IET can be obtained from the resultant electric field ***E***(***r***,*ω*) as

2 $$\frac{{dW}}{{dt}} = - \frac{1}{2}\Re \int{\boldsymbol{J}^\ast\cdot \boldsymbol{E} \, d^{3}r} = \frac{\omega }{2}\Im \left( {\boldsymbol{\mu} ^ {\ast} \cdot \boldsymbol{E}} \right)$$

where ***J***^∗^ and ***μ***^∗^ represents the complex conjugate of the source terms; and $\Re$ and $\Im$ respectively represent the real and imaginary parts. In the weak coupling limit, the inelastic transition decay rate *γ* associated with energy ℏ*ω* is $\gamma = \left( {\frac{1}{{\hbar \omega }}} \right)\frac{{dW}}{{dt}}$ and in terms of LDOS (*ρ*(***r***_**o**_,*ω*)), $\gamma = \left( {\frac{{\pi \omega }}{{\hbar {\it{\epsilon }}_0}}} \right)\left| {{{\boldsymbol{\mu }}}} \right|^2\rho \left( {{{{\boldsymbol{r}}}}_{{{\boldsymbol{o}}}},\omega } \right)$. The normalized transition decay rate ${\Gamma}\left( { = \frac{\gamma }{{\gamma _0}}} \right)$ can be obtained from the system’s dyadic Green’s function $\overleftrightarrow {\boldsymbol{G}}\left( \boldsymbol{r_0,r_0} \right)$ as:

3 $${\Gamma} = \frac{{6\pi c}}{\omega }\Im \left[ {\boldsymbol{n_\mu} \cdot \overleftrightarrow{\boldsymbol{G}}\left( \boldsymbol{r_0,r_0} \right)\boldsymbol{n_\mu}} \right]$$

where ***n***_***μ***_ represents the unit vector in the direction of ***μ***, *γ*_0_ gives the decay rate in free space, and Γ consists of radiative (Γ_R_) and non-radiative (Γ_NR_) contributions where Γ_R_ represents the photon emission from the TJ and Γ_NR_ contains nonradiative SPP excitation and the absorption losses in the electrodes. The dyadic Green’s function $\overleftrightarrow{\boldsymbol{G}}\left( \boldsymbol{r_0,r_0} \right)$ for the system can be calculated from the angular spectrum representation of the electric field for the dipole field as described in Supplementary Section 2 (See Eq. S34).

In the following, we consider the optic axis of the hBN parallel to *z*, along the tunneling direction (Fig. 1b). For an anisotropic medium ($\tilde \epsilon$) the electric displacement field $\boldsymbol{D} = \epsilon_{0}\widetilde{\epsilon}\boldsymbol{E}$, where $\tilde \epsilon$ represents the permittivity tensor^50^. The dispersion relations for the ordinary and extra-ordinary waves are given by $\left( {s_x^2 + s_y^2 + s_z^2} \right)/ \epsilon _{||} = 1$ and $\left( {s_x^2 + s_y^2} \right)/ \epsilon _ \bot + s_z^2/ \epsilon _{||} = 1$ respectively^50^, with *s*_*x*,*y*_ and *s*_*z*_ representing the in-plane and out-of-plane wave vectors respectively, normalized with free space wavenumber *k*_0_. For a dipole oriented along *z*, $dW/dt = \sqrt { \epsilon _\parallel } \left| \boldsymbol\mu \right|^2\omega k_0^3/(12\pi \epsilon _0)$^46^ and the normalized transition decay rate Γ’ for the anisotropic system can be obtained from the modified dyadic Green’s function $\overleftrightarrow {\boldsymbol{G^\prime}}\left( \boldsymbol{r_0,r_0} \right)$using Eq. (3). For calculating $\overleftrightarrow {\boldsymbol{G^\prime}}\left( \boldsymbol{r_0,r_0} \right)$ (see Eq. S34) for the anisotropic air-Au//hBN//Gr-glass system, reflected fields from each material interface are obtained for the individual plane waves in the angular spectrum as described in Supplementary Section 2.3. It should be noted that the normalization used here in calculating Γ’ is $\gamma _0^\prime = \sqrt { \epsilon _\parallel } \left| \boldsymbol\mu \right|^2k_0^3/\left( {12\pi \hbar \epsilon _0} \right)$, which represents the decay rate of a $\hat z$ oriented dipole in a homogeneous anisotropic medium represented by Eq. 1 (See Supplementary Section 2).

**Fig. 2:**
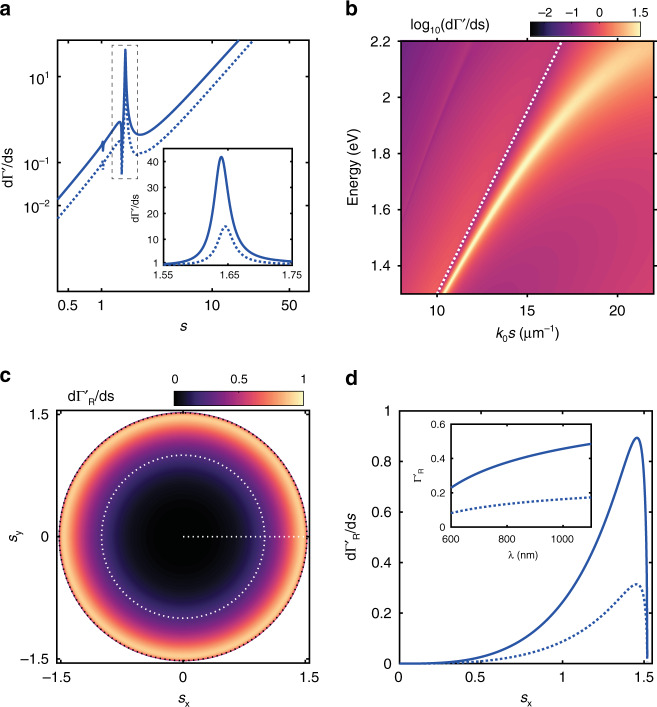
Radiative and non-radiative decay rates for the Au//hBN//Gr TJ. **a** Power dissipation spectrum for the anisotropic (*d*Γ’/*ds*, solid-line) and the isotropic (*d*Γ/*ds*, dotted-line) case evaluated at 750 nm for the Au//hBN//Gr TJ. The peaks at *s* ∼ 1.58 (inside the dashed-line box) represent SPP_glass_ mode. Inset shows the plot in linear scale, demonstrating the enhancement in *d*Γ'/*ds* (∼ ×4) as compared to *d*Γ/*ds*. **b** *d*Γ’/*d*s plotted as a function of *k*_0_*s* and $\hbar$*ω* (in log10 scale), representing the dispersion of the Au//hBN//Gr TJ. The bright band represents the dispersion of the SPP_glass_ mode and the dotted line shows the light dispersion in the glass. **c** Radiative decay rate spectrum $d{\Gamma}_{{{\mathrm{R}}}}^\prime /ds$ evaluated at *λ* = 750 nm as a function of (*s*_*x*_, *s*_*y*_). Light-lines in air $\left( {{s}_{x}^2 + {s}_{y}^2 = {1}}\right)$ and glass $( s_x^2 + s_y^2 = n_{\rm{glass}})$ media are shown as inner and outer dashed circles respectively. **d** *d*$\Gamma^\prime_{\mathrm R}$/*ds* (solid-line) obtained along the dashed-horizontal line in (**c**) and the corresponding *d*Γ_R_/*ds* (dotted-line) for the isotropic case, demonstrating the enhancement in $d\Gamma_{\mathrm R}^\prime/ds$(∼ ×3) as compared to *d*Γ_R_/*ds*. Inset shows the total radiative decay rate for the anisotropic ($\Gamma_{\mathrm R}^\prime$, solid-line) and the isotropic (Γ_R_, dotted-line) case evaluated in the wavelength range of 600-1100 nm

Fig. 2a shows the calculated power dissipation spectrum *d*Γ’/*ds* obtained from Γ’ as a function of $s\left( { = \sqrt {s_x^2 + s_y^2} } \right)$ for the Au//hBN//Gr junction (solid line) and is evaluated at ℏ*ω*≡750 nm. We used *ϵ*_∥_= 4.96 and *ϵ*_⊥_ = 2.98 for the anisotropic hBN layer^40^ and the dotted line shows power dissipation spectrum *d*Γ/*ds* for the isotropic case (*ϵ*_∥_ = *ϵ*_⊥_ = 4.96). The main peak at *s* ≈ 1.58 (inside the dashed-line box) represents the SPP mode, supported by the Au-interface in contact with the hBN-Gr-glass stack, and is denoted as SPP_glass_ (see Fig. 1b). The inset to Fig. 2a shows the *d*Γ’/*ds* and *d*Γ/*ds* corresponding to the SPP_glass_ peak plotted in the linear scale. By invoking the anisotropy for the hBN layer, the magnitude of *d*Γ’/*ds* is enhanced by a factor of 4 at *s* ≈ 1.58, compared to *d*Γ/*ds*. The minor peaks at *s* ≈ 1 represent the SPP mode supported by the Au-air interface. In the calculation, we used 60 nm thick Au film, which is approximately twice the skin depth of Au. Yet a minor, but distinguishable dipole power dissipation for the Au-air SPP mode is visible, which is evident in the log-scale (*y*-axis) representation. Fig. 2b shows the dispersion relation for the Au//hBN//Gr TJ, obtained by calculating *d*Γ'/*ds* as a function of $k_0s$ and ℏ*ω*. The bright-band represents the dispersion of the SPP_glass_ mode which lies at the right-hand side of the light-line (dotted line) in the glass medium, signifying the bounded nature of the mode.

Next, we investigate the effect of the optical anisotropy of the hBN on the direct light emission from the TJ by calculating the radiative decay rate ${\Gamma}_{{{\mathrm{R}}}}^\prime$ $\left( {\left[ {1/\gamma _0^\prime } \right]{\int} {d\boldsymbol{A} \, \langle\frac{1}{2} \, \boldsymbol{E} \times \boldsymbol{H}^ \ast }\rangle } \right)$ of the dipole^52^ from the electric (***E***'(***r***,***r***_**0**_,*ω*)) and magnetic (***H***'(***r***,***r***_**0**_,*ω*)) field vectors as described in the Supplementary Section 3. Fig. 2c shows the heatmap of $d{\Gamma}_{{{\mathrm{R}}}}^\prime /ds$ evaluated at 750 nm as a function of *s*_*x*_ and *s*_*y*_, representing the direct light emission from the TJ to the far-field through the glass medium. The inner dashed-circle represents the critical angle of the air-glass interface (*s* = 1) and the outer dashed-circle represents *s* = *n*_glass_ = 1.52. The intensity distribution in Fig. 2c shows that majority of the radiation outcoupling is directed towards *s*≈ 1.52, representing emission into the glass substrate at high angles. To compare the radiative decay rate between the isotropic and anisotropic cases, we evaluate $d{\Gamma}_{{{\mathrm{R}}}}^\prime {{{\mathrm{/}}}}ds$ and *d*Γ_R_/*ds* along the dashed-horizontal line shown in Fig. 2c which are plotted in Fig. 2d. As noted before, both $d{\Gamma}_{{{\mathrm{R}}}}^\prime /ds$ (solid-line) and *d*Γ_R_/*ds* (dotted-line) have dominant contributions for *s*_*x*_≥ 1. For $d{\Gamma}_{{{\mathrm{R}}}}^\prime /ds$, however, we observe a factor of ∼ 3 enhancement around *s*_*x*_≈ 1.51 relative to *d*Γ_R_/*ds* which is consistently observed in the wavelength range 600–1100 nm as shown in the inset to Fig. 2d, where ${\Gamma}_{{{\mathrm{R}}}}^\prime$ (solid-line) and Γ_R_ (dashed line) obtained from integrating $d{\Gamma}_{{{\mathrm{R}}}}^\prime /ds$ and *d*Γ_R_/*ds* over *s* from 0 to 1.52 are plotted.

**Fig. 3:**
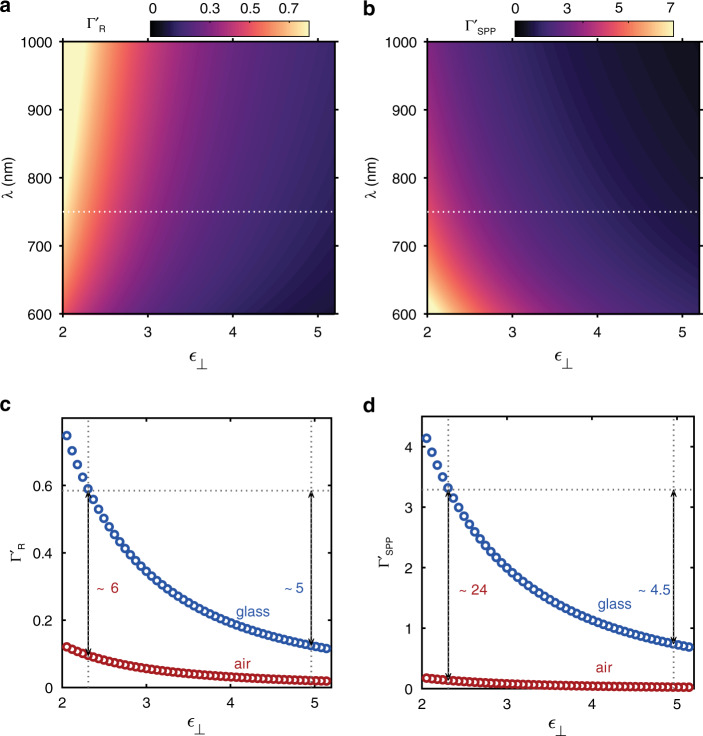
Decay rate for a general uniaxial medium. **a**, **b** ${\Gamma}_{{{\mathrm{R}}}}^\prime$ and ${\Gamma}_{{{{\mathrm{SPP}}}}}^\prime$ evaluated in the wavelength range of 600–1100 nm as a function of the *ϵ*_⊥_. **c**, **d** ${\Gamma}_{{{\mathrm{R}}}}^\prime$ and ${\Gamma}_{{{{\mathrm{SPP}}}}}^\prime$ evaluated along the horizontal-dotted lines in (**a**, **b**) corresponding to *λ* = 750 nm (blue circles). Red circles represent the data for ${\Gamma}_{{{\mathrm{R}}}}^\prime$ and ${\Gamma}_{{{{\mathrm{SPP}}}}}^\prime$ evaluated for the TJ geometry with glass replaced by air. Vertical-dotted lines in (**c**) and (**d**) represent $\epsilon _ \bot = n_{\rm{glass}}^2 ={2.31}$ (left) and *ϵ*_⊥_ = *ϵ*_||_ = 4.96 (right; isotropic case). Right vertical arrows demarcate the enhancement in ${\Gamma}_{{{\mathrm{R}}}}^\prime$ and ${\Gamma}_{{{{\mathrm{SPP}}}}}^\prime$ for the isotropic to anisotropic transition and the left vertical arrows give the enhancement at *ϵ*_⊥_ = 2.31 with respect to the air case

From the previous discussion, it is evident that the anisotropic nature of the hBN layer enhances the radiative $\left( {d{\Gamma}_{{{\mathrm{R}}}}^\prime /ds} \right)$ and nonradiative (*d*Γ'/*ds*) LDOS associated with the IET mediated SPP excitation and light emission from the TJ. For a quantitative analysis of the enhancement factor, we calculate the decay rate for a general uniaxial medium with *ϵ*_⊥_ = *ϵ*_*||*_±Δ*ϵ*. Here, Δ*ϵ* denotes the anisotropy parameter and the condition Δ*ϵ* = 0 corresponds to the isotropic case (*ϵ*_⊥_ = *ϵ*_*||*_ = 4.96 for hBN^40^). The radiative decay rate for the photons ${\Gamma}_{{{\mathrm{R}}}}^\prime$ and the nonradiative decay rate for the SPPs ${\Gamma}_{{{{\mathrm{SPP}}}}}^\prime$ are calculated as a function of Δ*ϵ* with *λ* = 600–1100 nm. Fig. 3a and b respectively show ${\Gamma}_{{{\mathrm{R}}}}^\prime$ and ${\Gamma}_{{{{\mathrm{SPP}}}}}^\prime$calculated as a function of *ϵ*_⊥_ (= *ϵ*_||_± Δ*ϵ*) and *λ*. Both ${\Gamma}_{{{\mathrm{R}}}}^\prime$ and ${\Gamma}_{{{{\mathrm{SPP}}}}}^\prime$monotonically increases with decrease in *ϵ*_⊥_. We note that an increase in ${\Gamma}_{{{{\mathrm{SPP}}}}}^\prime$ towards *λ*≈ 600 nm is also observed, which we attribute to the onset of interband damping (∼ 2 eV) in the Au electrode. Fig. 3c and d respectively show ${\Gamma}_{{{\mathrm{R}}}}^\prime$ and ${\Gamma}_{{{{\mathrm{SPP}}}}}^\prime$ evaluated along the dotted lines (*λ* = 750 nm) in Fig. 3a and b. With the decrease in *ϵ*_⊥_ from 4.96 (right vertical dotted line in Fig. 3c and d) to *ϵ*_⊥_=2.31 ($n_{glass}^2$; the left vertical dotted line in Fig. 3c and d) a monotonic rise is observed for ${\Gamma}_{{{\mathrm{R}}}}^\prime$ (Fig. 3c) and ${\Gamma}_{{{{\mathrm{SPP}}}}}^\prime$ (Fig. 3d). The effect of the Gr layer is neglected in calculating ${\Gamma}_{{{\mathrm{R}}}}^\prime$ since Gr is transparent to the outcoupled radiation from the junction.^53^ In contrast to the isotropic case (*ϵ*_⊥_ = *ϵ*_||_ = 4.96; the right vertical dotted line in Fig. 3c and d), ${\Gamma}_{{{\mathrm{R}}}}^\prime$shows an enhancement factor of ≈ 5 for the anisotropic case (blue circles) when *ϵ*_⊥_matches the permittivity of the glass substrate (*ϵ*_⊥_ = 2.31). Fig. 3d shows a similar enhancement factor for ${\Gamma}_{{{{\mathrm{SPP}}}}}^\prime$ but with a lower rate in the transition from *ϵ*_⊥_ = 4.96 to *ϵ*_⊥_ = 2.31 than ${\Gamma}_{{{\mathrm{R}}}}^\prime$. Compared to the glass substrate in our case, air is the adjacent medium to the anisotropic hBN//Gr stack in the work of Parzefall et al.^33^. For comparing these two junction configurations, we calculated ${\Gamma}_{{{\mathrm{R}}}}^\prime$ and ${\Gamma}_{{{{\mathrm{SPP}}}}}^\prime$ (red circles Fig. 3c and d) for Au//hBN//Gr configuration with glass replaced by air. It is interesting to observe that the change in the substrate refractive index from 1 to 1.5 (air to glass) leads to an enhancement factor of ≈ 6 and ≈ 24 respectively for ${\Gamma}_{{{\mathrm{R}}}}^\prime$ and ${\Gamma}_{{{{\mathrm{SPP}}}}}^\prime$ at *ϵ*_⊥_ = 2.31. This is similar to the observed radiative and nonradiative enhancement for the quantum emitters by the presence of high index substrates^53^. The above results summarize that the transition from an isotropic to an anisotropic case (decrease in *ϵ*_⊥_ from the right to the left dotted-vertical line in Fig. 3c and d) induces an enhancement in both ${\Gamma}_{{{\mathrm{R}}}}^\prime$ and ${\Gamma}_{{{{\mathrm{SPP}}}}}^\prime$ by a factor of ≈ 5 and ≈ 4.5 respectively. This observed enhancement can be attributed to the increase in LDOS, originating from the elliptic dispersion of the anisotropic medium^50^. This is reflected in the scaling of the dipole field amplitude with the anisotropy^54^ as the vector potential ***A***(***r***) of the dipole field scales with the ratio *ϵ*_*||*_/*ϵ*_⊥_(See Eq. S39, Supplementary Section 2)^46^ and results in the observed enhancement with decrease in *ϵ*_⊥_, for a constant $\epsilon_{||}$.

Next, we discuss the experimental investigation of the photon emission from the Au//hBN//Gr TJ to the far-field through the glass substrate, concomitant to the direct SPP and photon excitation, using the real-plane and the BFP imaging. From the previous discussion on ${\Gamma}_{{{\mathrm{R}}}}^\prime$, a high index (glass) substrate plays a crucial role in radiation outcoupling from the dipolar emitter embedded in an anisotropic hBN layer adjacent to the substrate. Therefore, in the following, we perform a systematic analysis of the experimental emission intensity through the glass substrate based on the theoretical model for the dipole decay in an anisotropic medium.

**Fig. 4:**
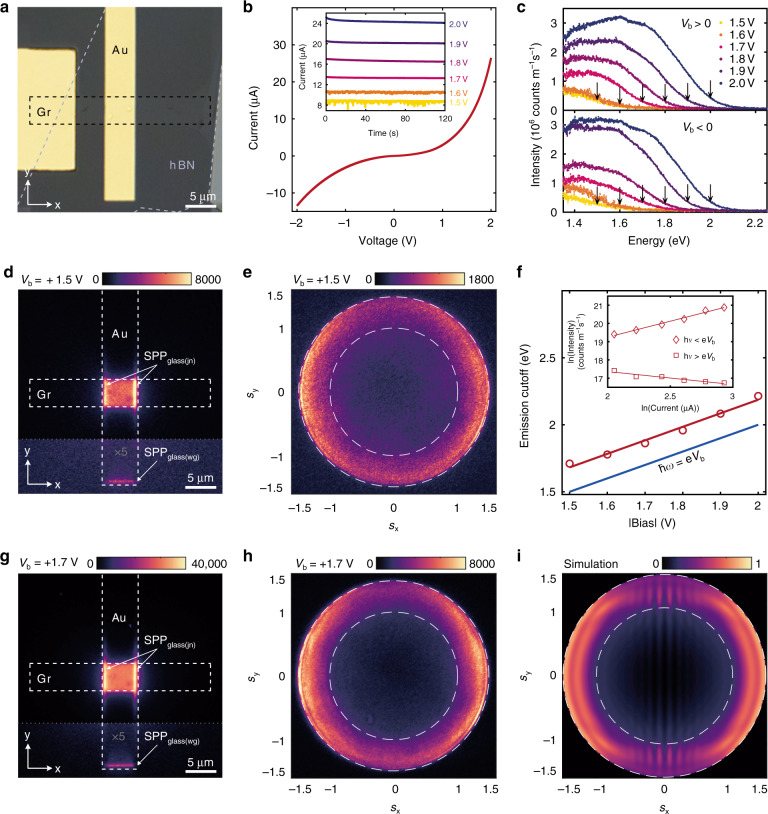
Electrical and optical characteristics of the Au//hBN//Gr TJ. **a** Optical image of the fabricated Au//hBN//Gr TJ. Dashed lines represent the outline of Gr (black) and hBN (gray) layers. **b** Measured current-voltage (*I-V*) characteristics of the device. Inset shows the *I*(*t*) traces representing current values recorded over 120 s for a constant bias in the range of 1.5 to 2.0 V. **c** Light emission spectra for positive (top) and negative (bottom) bias (*V*_b_) in the range of 1.5 to 2.0 V, collected over 120 s. Downward arrows indicate the theoretical energy quantum cutoff ℏ*ω*=|e*V*_b_|. Spectral intensity is corrected for the detection efficiency of the optical system (see Supplementary Fig. S4). **f** Maximum emission energy (emission cutoff) of the experimental spectra shown in (**c**), averaged over +*V*_b_ and –*V*_b_ (open circle) and the linear fit (red solid line). The Blue solid line represents ℏ*ω* = |e*V*_b_|. Inset shows the emission intensity integrated below (open rhomb) and above (open square) the theoretical quantum cutoff and the solid lines are power-law fit *I*^*β*^ to the data (see the text). Real plane ((**d**) and (**g**)) and BFP ((**e**) and (**h**)) images of the light emission from the TJ at two representative bias voltages (1.5 V and 1.7 V), for which a spatially localized intensity enhancement is distinguishable at the right and left edges of the TJ (*x*-direction). For the BFP images, the inner dotted-circle refers to *s* = 1 and the outer dotted-circle corresponds to NA of the objective, *s* = 1.49. **i** BFP image for the radiation outcoupling from a 5 × 5 μm^2^ Au//hBN//Gr TJ simulated using finite difference time domain method. Intensity scales in real and BFP images are adjusted to give the best possible contrast for the emission features

Fig. 4a shows the optical microscope image of the fabricated device, where a few-layer Gr (∼ 1.0 nm thick) strip (5 μm wide) defined by lithography and plasma etching serves as the electrode, and an Au (5 μm wide) strip with a thickness of 60 nm serves as the counter electrode and a plasmonic waveguide. The overlapping region of the two electrodes defines the junction area. A 7-layer hBN (∼ 2.3 nm thick) electrically separates the Gr and Au electrodes. This ensures a stable tunnel current (∼ few μA) within the breakdown limit for low applied bias values (< 2.5 V). See Supplementary Section 5 for the fabrication details and the AFM images of the device. Apart from PMMA residues left from the transfer process, the interfaces are smooth and conformal resulting in uniform light emission from the junction area.

To characterize the TJ, we performed electrical and optical measurements as described in Supplementary Section 6. The TJ is biased according to the layout shown in Fig. 1a. Fig. 4b shows the typical nonlinear current-voltage (*I-V*) plot obtained for the TJ. The inset to Fig. 4b shows the current-time traces *I*(*t*) recorded over 120 s for a given bias *V*_b_ in the range 1.5 to 2.0 V, demonstrating a stable tunneling in the spectral integration time window (120 s). The few-layer graphene electrode used in our device maintains good contact with the hBN layer due to the atomic flatness and the uniform van der Waals contact^55^ judged from the uniform light emission from our junctions. The currents (on the order of μA μm^−2^) across our junctions are similar to those reported with a similar device structure^33^, such large currents are straightforward to measure in a Keithly 6430 sub-femto amp electrometer (See Materials and Methods). Fig. 4c shows the emission spectra of the TJ for *V*_b_ in the range 1.5–2.0 V for +*V*_b_ (top) and -*V*_b_ (bottom). With respect to the bias polarity (±*V*_b_), the spectra show identical response in terms of the position and the blue shift of the peaks with increasing |*V*_b_|, consistent with the IET mediated light emission characteristics^14^. Fig. 4f shows the maximum emission energy (emission cutoff) averaged over ± *V*_b_ and the red solid line represents a linear fit to the data. The blue solid line in Fig. 4f represents the theoretical quantum cutoff ℏ*ω* = |e*V*_b_|, from which the emission cutoff shows a constant offset (∼+ 100 meV) across the whole bias window, indicating that the light emission has contributions from energies beyond the theoretical cutoff. The emission intensity integrated below and above the quantum cutoff is plotted as a function of 〈*I*(*t*)〉 in the inset to Fig. 4f, where 〈*I*(*t*)〉 represents the time average of *I*(*t*) obtained from Fig. 4b-inset (averaged over +*V*_b_ and –*V*_b_). The solid lines represent a power-law fit *I*^*β*^. Intensity above the cutoff shows a nearly tunnel current independent emission, which we attribute to the thermal broadening of the emission and rule out any higher-order electron-plasmon interactions^56^ involved in the above cutoff emission. The emission below the quantum cutoff follows the power law with *β* ∼ 1.7, indicating a super-linear dependence of the light emission intensity on the tunnel current (see Supplementary Section 7 for the spectral cutoff calculations).

Fig. 4d and g show the real plane and Fig. 4e and h show the Fourier-plane images of the far-field emission from the TJ, for two representative bias voltages 1.5 and 1.7 V (See Supplementary Section 6 for the full data set). We make the following observations from the real plane images shown in Fig. 4d and g: Uniform light emission is observed across the TJ area (5×5 μm^2^) with a spatially localized intensity enhancement at the right and left edges of the TJ (*x*-direction). Since Gr is optically transparent, photons collected in the far-field originate either from the direct emission or from the radiative decay of the SPPs traveling in the *x*-direction and scattering to the far-field from the right and left edges of the TJ (SPP_glass(jn)_ in Fig. 4d and g). This is in stark contrast with the SPPs traveling in the *y*-direction along the Au waveguide, which does not experience any discontinuity at the top and bottom edges of the TJ. Instead, these SPPs travel along the Au waveguide and undergo scattering at the waveguide-end (*-y* direction; SPP_glass(wg)_ in Fig. 4d and g) resulting in comparatively weak emission intensity (color scale is rescaled (×5) for better contrast). For plasmonic MIM-TJs, a highly confined MIM-SPP mode with a high effective index (*s* ∼ 10) is present and therefore is crucially affected by the small-scale roughness (∼ (*k*_0_*s*)^-1^) of the top and bottom electrodes, leading to the roughness mediated light emission from MIM-TJs^20^. However, for the Au//hBN//Gr junction, a MIM-SPP mode is absent as the mode supported by the junction has single-interface SPP characteristics (SPP_glass_) with *s* ∼ 1.58 (Fig. 2a) and therefore remains insensitive to the small-scale roughness of the Au electrode. This observation helps us to attribute the uniform light emission from the TJ area to the direct light emission from the Au//hBN//Gr TJ and to rule out any roughness mediated light emission from the TJ, as the entire Au waveguide remains dark, but at the TJ-area and the waveguide-end^33^. Therefore, we conclude that the light emission observed from TJ-area is dominated by the photons and SPPs directly excited via IET.

We analyzed the BFP images (Fig. 4e and h) for the light emission from the TJ to further confirm the above observations. These images show a diffuse annular intensity distribution between the inner (*s* = 1) and outer (*s* = 1.49) dotted circles resembling the radiation pattern of a vertical dipole close to the metal surface^33^, consistent with the theoretical results shown in Fig. 2c with the majority of the light emission (either direct or SPP mediated) directed towards the high-angles through the glass substrate. The two symmetrical bright lobes in the ±*s*_*x*_ direction around *s* ≈ 1.49 (Fig. 4e and h) correspond to the SPP_glass_ scattering from the right and left edges of the TJ (see Supplementary Section 10). Fig. 4i shows the BFP image for the radiation outcoupling from a 5×5 μm^2^ Au//hBN//Gr TJ simulated using a finite difference time domain model which confirms this interpretation.

## Discussion

**Fig. 5:**
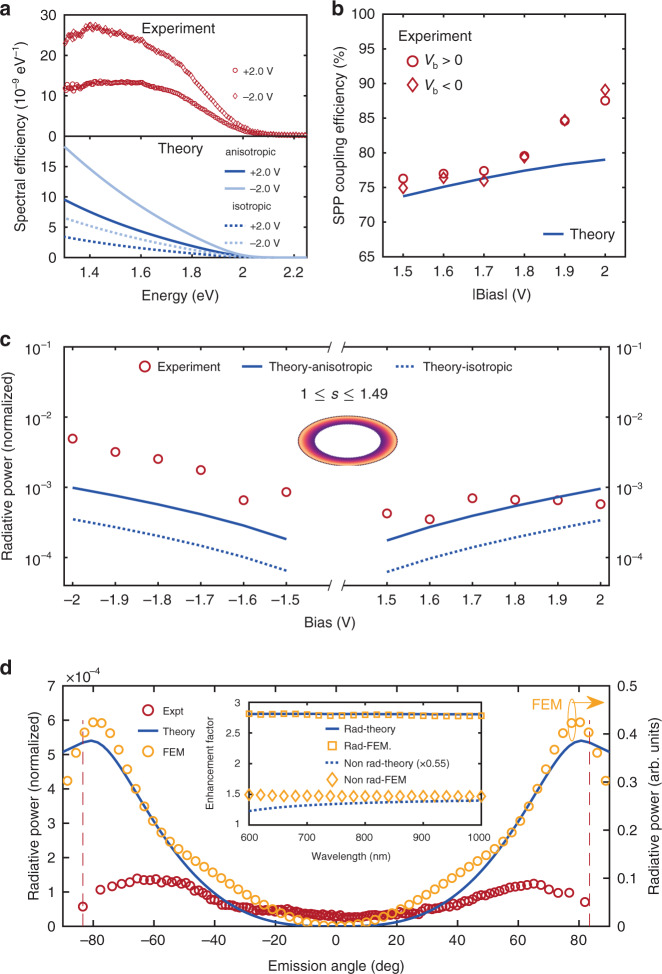
Theory-experiment comparison quantifying the role of the hBN anisotropy. **a** Top panel: Spectral efficiency (number of photons per tunneling electrons) of the junction from the experiment (top) for *V*_b_ = +2.0 V (open circle) and for –2.0 V (open rhomb). Bottom panel: Spectral efficiency calculated from theory for *V*_b_ = +2.0 V (dark blue) and for –2.0 V (light blue). Solid and dotted lines correspond to anisotropic and isotropic cases respectively. **b** Relative coupling efficiency for SPP excitation obtained as a function of |*V*_b_| from the experiment (for *V*_b_> 0 (open circle) and *V*_b_< 0 (open rhomb)) and from theory (solid line). **c** Normalized radiative power from the junction, evaluated as a function of ±*V*_b_. Inset shows the BFP angular emission range (1 ≤ *s* ≤ 1.49) where the radiative power is integrated (see text). **d** Comparison between the theory, experiment, and the FEM simulations. Normalized radiative power for *V*_b_ = 2.0 V, evaluated as a function of emission angle. The left *y*-axis shows the results from the theory (anisotropic case, blue solid line) and the experiment (red circles), and the radiative power obtained from the FEM simulation is shown on the right *y*-axis (orange circles). Dashed vertical lines represent the angle corresponding to the numerical aperture of the objective. Inset shows the enhancement of the radiative (FEM (open square), theory (solid line)) and non-radiative decay rate (FEM (open rhomb), theory (dotted line)) for the anisotropic to the isotropic case

The spectral efficiency (number of photons emitted per tunneling electron) of the TJ is calculated from the emission spectra shown in Fig. 4c. In Fig. 5a spectral efficiency from the experiment (top panel) is compared with the theory (bottom panel) for *V*_b_ = ±2.0 V (see Supplementary Section 8 and 9 for details). Below the quantum cutoff (|*V*_b_| = 2.0 V), the spectral efficiency varies approximately between 0−10 × 10^−9^ per eV for *V*_b_ = +2.0 V (open circle), consistent with the anisotropic model (dark blue line). For *V*_b_ = –2.0 V (open rhomb), however, the spectral efficiency is underestimated from the theory (maximum of ∼ 18 × 10^−9^ per eV, light-blue line) compared to the experimental maximum of ∼ 27 × 10^−9^ per eV, which is attributed to the asymmetry in 〈*I*(*t*)〉 with ±*V*_b_ (See Fig. S3). Theoretical results from the isotropic model (dotted lines) underestimate the spectral efficiency in all cases, corroborating the enhancement associated with the hBN anisotropy. As explicitly shown in Fig. 3c, the presence of the glass substrate (*n* = 1.52) adjacent to the anisotropic hBN//Gr stack crucially affects the radiative outcoupling. To further corroborate this argument and to compare the theory with similar anisotropic systems from the past, we calculated the spectral efficiency for Au//hBN//Gr TJ with glass replaced by air medium (*n* = 1), similar to the junction geometry in Parzefall et al.^33^ (see Supplementary Fig. S8). The spectral efficiency decreases by a factor of ∼ 6, as estimated in Fig. 3c, and is consistent with the spectral efficiency reported by Parzefall et al. (< 2 × 10^−9^ per eV)^33^. Similar to the photon emission efficiency, we calculated the relative coupling efficiency of the SPP_glass_ mode (Π_SPP_) compared to the total power dissipated into the photonic and SPP_glass_ modes from the TJ (see Supplementary Section 8 for details). Fig. 5b shows Π_SPP_ evaluated as a function of |*V*_b_|. It is observed that, between the bias range of 1.5 V to 2.0 V, average Π_SPP_ can be as high as ∼ 80% (circles and rhombs), consistent with the theoretical estimate of ∼ 76% (solid line, Fig. 5b). This implies that the SPP decay channel is relatively stronger than the photonic outcoupling, essentially due to the higher LDOS associated with SPP_glass_ mode $\left( {{\Gamma}_{{{{\mathrm{SPP}}}}}^\prime } \right)$ than $$\begin{array}{l}{\Gamma}_{{{\mathrm{R}}}}^\prime \\ \end{array}$$ (see Fig. 2a, b).

To further elucidate the significance of the optical anisotropy in the direct light emission and SPP excitation, normalized radiative power from the TJ is calculated from the experimental BFP images and compared with the theoretical results. Fig. 5c shows the normalized radiative power evaluated as a function of ±*V*_b_. The radiative power is calculated by integrating the emitted power within the range 1 ≤ *s* ≤ 1.49 (inset to Fig. 5c). For a meaningful comparison, current dipole amplitude |***μ***| (in units of A.m) calculated from〈*I*(*t*)〉(Fig. 4b inset) is used to normalize the integrated ${\Gamma}_{{{\mathrm{R}}}}^\prime$ (solid line) and Γ_R_ (dotted line) from the theory (see Supplementary Section 8). The normalized radiative power from the experiment is in qualitative agreement with the anisotropic model with an observed asymmetry with respect to ±*V*_b_ in comparison with the theory. Fig. 5d shows the normalized radiative power for the light emission from the junction area (for *V*_b_ = 2.0 V), evaluated as a function of emission angle (sin^−1^(*s*/*n*_glass_)). The left y-axis represents the results from the anisotropic case (blue solid line) and the experiment (red circles). The radiative power obtained from a finite element method (FEM) simulation of the TJ is plotted on the right *y*-axis (orange circles) and shows a consistent agreement with the theoretical and experimental radiative power as a function of the emission angle. Furthermore, inset to Fig. 5d shows the ratio ${\Gamma}_{{{\mathrm{R}}}}^\prime /{\Gamma}_R$, which represents the enhancement factor for the radiative decay rate for the anisotropic case related to the isotropic, evaluated for *λ* from 600 to 1000 nm. Theory (solid line) and the FEM (open squares) results show excellent agreement. An enhancement factor of ∼ 3 is observed for the anisotropic case compared to the isotropic system, across the entire *λ* range. However, the non-radiative decay rate calculated from the theory shows a discrepancy by a factor of 0.55 compared to the FEM result, which can be attributed to the limited spatial extent of the simulation domain in FEM, limiting the contributions from single interface SPPs (See Supplementary Section 11).

## Conclusions

To summarize, we demonstrate direct SPP excitation and photon emission from an Au//hBN//Gr TJ. By carrying out a rigorous theoretical analysis for the IET process using a dipole embedded in a uniaxial medium, we identified the role of the anisotropy of hBN on the radiative and nonradiative LDOS associated with IET. Though Gr forms an integral part of the TJ and sustains uniform tunnel current across the TJ-area, it is plasmonically inactive in the visible and near-IR energy range. Since, for IET, most of the energy transfer process is localized to the immediate vicinity of the TJ, it is expected that the local dielectric function around TJ will crucially affect the LDOS. We theoretically demonstrate this hypothesis by showing that with a change in *ϵ*_⊥_ from ∼ 5 to ∼ 3 associated with a 2.3 nm thick hBN insulating layer, the radiative and nonradiative decay rates are enhanced by a factor of ∼ 3. Although Gr-hBN-plasmonic metal TJs have been demonstrated before^33–35^, the effect of the layered nature of hBN leading to the uniaxial anisotropy has not been investigated. The theoretical treatment presented here extends the dipole embedded in an anisotropic medium^45^ model to the case of an IET mediated excitation of plasmons and photons which is highly pertinent for device designs involving plasmonic-2D material hybrid systems.

Experimentally we observe uniform light emission and SPP excitation from the TJ-area consistent with the theoretical treatment. We show that the device configuration of Au//hBN//Gr TJ with glass as the adjacent medium to the anisotropic hBN//Gr heterostructure leads to significant improvement (∼ ×3) in the photon emission rate per tunneling electron, compared to the previous report^33^. With the help of a dedicated SPP waveguide, we estimate the relative SPP coupling efficiency as ∼ 80% stronger than the radiative contribution, adding an important insight to the 2D material-metal hybrid systems as electrically driven SPP sources for on-chip integration. This is in stark contrast to typical MIM-TJs^20^, where excitation of a highly confined MIM-SPP mode with a large LDOS (10^4^−10^5^) associated with IET is involved, with stringent momentum matching requirements between the MIM-SPP modes and various outcoupling pathways^21^. However, the direct interaction of the inelastic tunneling electrons with the photonic and plasmonic continuum demonstrated here offers an interesting platform with independent control over the light-matter interaction at the nanoscale, without the necessity to satisfy these requirements. Moreover, the direct method demonstrated here for the excitation of SPPs and photons from the open plasmonic system, helps to circumvent or minimize the plasmonic losses associated with the highly confined MIM-SPP mode associated with the MIM-TJs. Finally, we note that, in light of the recent advancements in 2D material integration with plasmonic systems^37^, the effect of the optical anisotropy considered in this work is one of the several factors to be investigated, especially in connection with IET, including nonlocality^57,58^ of the TJ dielectric properties.

## Materials and methods

### Numerical calculations

Radiative and nonradiative decay rates are numerically calculated using Matlab, based on the theory discussed in Supplementary Sections 1–3. Interpolated data sets are used to represent the permittivity of Au^49^ and Gr^51^ electrodes in numerical calculations. Finite element simulations were carried out with COMSOL Multiphysics 5.1. Full-field electromagnetic simulations were performed with the wave optics module for the electric point dipole located in the anisotropic hBN layer. Time-averaged Poynting vector integrated over the simulation boundaries gives the radiated power and the nonradiative energy loss rate was calculated by integrating the total power dissipation density over the electrode domains. Perfectly matched layers were used as simulation boundaries.

### Fabrication

The Au//hBN//Gr TJ device was fabricated on a glass coverslip (Marienfield, 160 μm thick). The graphene (∼ 1.0 nm, corresponding to 3 layers) and hBN (∼ 2.3 nm, corresponding to 7 layers) flakes were exfoliated on a PMGI/PMMA double sacrificial layer, from commercial natural graphite (NGS Naturagraphit GmBH) and hBN (HQ Graphene) crystals respectively. We dissolved the PMGI layer with MF319 and float the PMMA layer carrying flakes. The PMMA layer was removed from the flakes in acetone after transfer. The graphene flake was patterned as a 5 μm wide strip using O_2_ plasma (Femto Science, VITA) etching with a PMMA mask fabricated with electron beam lithography (EBL, JEOL, JBX-6300FS). The hBN flake was then stacked on the graphene strip. The hBN-Gr assembly was annealed in a vacuum at 250 °C for 12 h for a better interlayer and flakes-substrate contacts. We then confirmed the flake thickness using AFM. The 5 μm wide Ti/Au (1 nm/60 nm) electrode was fabricated using EBL and thermal evaporation (Kurt J. Lesker, NANO 36).

### Electrical and optical characterization

The device was characterized using an inverted optical microscope (Nikon, Eclipse Ti-E) equipped with an EMCCD (Andor, iXon Ultra 897) and a spectrometer (Andor, Shamrock 303i). The device emissions were collected from the backside of the substrates with an oil-immersed objective (Nikon, 100×, NA 1.49), when the devices were biased using a source meter (Keithley 6430). Fourier-plane images were captured by projecting the back focal plane of the objective on the EMCCD using a Bertrand lens. Spectral data are corrected for the detection efficiency of the optical system (see Supplementary Fig. S4).

## Supplementary information

Supplementary materials

Graphical abstract

## Data Availability

The data that support the findings of this study are available from the corresponding author upon reasonable request.
