# Supplementary material for: Optical Anisotropy in van der Waals materials: Impact on Direct Excitation of Plasmons and Photons by Quantum Tunneling
Source: Light Sci Appl. 2021 Nov 8;10:230. doi: 10.1038/s41377-021-00659-7 (PMC8575904; doi:10.1038/s41377-021-00659-7)
Supplement: Supplementary file 1 — Supplementary materials [file 41377_2021_659_MOESM1_ESM.docx]

**SUPPORTING INFORMATION**

Optical Anisotropy in van der Waals materials: Impact on Direct Excitation of Plasmons and Photons by Quantum Tunneling

*Zhe Wang^†, 1^, Vijith Kalathingal**^, †,^ *^1,2,3^, Thanh Xuan Hoang^4^, Chu Hong Son^4^, and Christian A. Nijhuis**^,^*^1,2,5^*

^1^Department of Chemistry, National University of Singapore, 3 Science Drive 3, Singapore 117543, Singapore

^2^Centre for Advanced 2D Materials, National University of Singapore, 6 Science Drive 2, Singapore 117564, Singapore

^3^Department of Electrical and Computer Engineering, National University of Singapore, 4 Engineering Drive 3, 117583, Singapore

^4^Department of Electronics and Photonics, Institute of High Performance Computing, A*STAR (Agency for Science, Technology and Research), 1 Fusionopolis Way, Singapore 138632, Singapore

^5^Hybrid Materials for Opto-Electronics Group, Department of Molecules and Materials, MESA+ Institute for Nanotechnology and Center for Brain-Inspired Nano Systems, Faculty of Science and Technology, University of Twente, 7500 AE Enschede, The Netherlands

^†^ These authors contributed equally to this work.

*Authors to whom correspondence should be addressed: c.a.nijhuis@utwente.nl, vijith.k@nus.edu.sg

**S1. Reflection and transmission coefficients**

The Maxwell’s equations in a non-magnetic and source-free region are:

$\boldsymbol{\nabla}\cdot\boldsymbol{D}=0$ (S1)

$\boldsymbol{\nabla}\cdot\boldsymbol{B}=0$ (S2)

$\boldsymbol{\nabla}\times\boldsymbol{E}=-\frac{\partial\boldsymbol{B}}{\partial t}$ (S3)

$\boldsymbol{\nabla}\times\boldsymbol{H}=-\frac{\partial\boldsymbol{D}}{\partial t}$ (S4)

In the time harmonic limit for the plane wave solutions, $\partial/\partial t\to-i\omega$, $\boldsymbol{\nabla}\times\to i\boldsymbol{k}\times$, $\boldsymbol{\nabla}\cdot\to i\boldsymbol{k.}$ For an anisotropic medium, constitutive relations between electric and magnetic fluxes with the electric and magnetic fields are (we refer to Chew^1^ for a general discussion):

$\boldsymbol{D}=\epsilon_{0}\bar{\boldsymbol{\epsilon}}\boldsymbol{E}$ (S5)

$\boldsymbol{B}=\mu_{0}\boldsymbol{H}$ (S6)

For a lossless anisotropic medium, the permittivity tensor $\bar{\boldsymbol{\epsilon}}$ will be Hermitian $\epsilon_{ij}=\epsilon_{ij}^{*}$^2,3^. Following Chew^1^, for a homogeneous, anisotropic medium, both type-I (ordinary) and type-II (extra-ordinary) waves exist and in general they are decoupled from each other. In the presence of a planar interface, however, type-I may generate transmitted and reflected waves of type-I and type-II or vice versa. In other words, they will be coupled with each other. This is in stark contrast with transverse electric (TE) and transverse magnetic (TM) modes of a homogeneous isotropic medium where the two types of waves are still decoupled at the interface. For the non-magnetic case, equations S1-S4 become:

$\boldsymbol{k}\cdot\left( \bar{\boldsymbol{\epsilon}}\boldsymbol{E} \right)=0$ (S7)

$\boldsymbol{k}\cdot\boldsymbol{H}=0$ (S8)

$\boldsymbol{k}\times\boldsymbol{E}=\omega\mu_{0}\boldsymbol{H}$ (S9)

$\boldsymbol{k}\times\boldsymbol{H}=-\omega\epsilon_{0}\bar{\boldsymbol{\epsilon}}\boldsymbol{E}$ (S10)

The last two equations can be combined to obtain:

$\left( \boldsymbol{k}\cdot\boldsymbol{E} \right)\boldsymbol{k}-k^{2}\boldsymbol{E}\boldsymbol{+}k_{0}^{2}\bar{\boldsymbol{\epsilon}}\boldsymbol{E}=0$ (S11)

Note that for an isotropic medium, the first term of Eq. S11 will be zero, followed from Eq. S1. For an anisotropic system, however, $\boldsymbol{E}$and $\boldsymbol{D}$ are no longer parallel to each other so that ($\boldsymbol{k}\cdot\boldsymbol{E}$) will not vanish. For an optically anisotropic system, in general, three principal dielectric components ($\epsilon_{x}, \epsilon_{y}, \epsilon_{z}$) of the medium are distinct with $\epsilon_{x}\neq\epsilon_{y}\neq\epsilon_{z}$and referred to as optically biaxial media. When $\epsilon_{x}=\epsilon_{y}=\epsilon_{\parallel}$ and $\epsilon_{z}=\epsilon_{\perp}$, the medium is called optically uniaxial, and the principal direction $\hat{z}$ coincides with the symmetry axis of the medium. For the uniaxial anisotropic system $\bar{\boldsymbol{\epsilon}}$ will be diagonal, as given by:

$\bar{\boldsymbol{\epsilon}}=\left( \begin{matrix} \epsilon_{\parallel} & 0 & 0 \\ 0 & \epsilon_{\parallel} & 0 \\ 0 & 0 & \epsilon_{\perp} \end{matrix} \right)$ (S12)

To simplify the formulation in the local density of states (LDOS) calculations, we consider only the uniaxial anisotropic system with $\hat{z}$ axis coinciding with the optical axis of the anisotropic medium. In the present description, both $\epsilon_{\parallel}$ and $\epsilon_{\perp}$are considered as positive and non-absorbing. The in-plane wave vector $\boldsymbol{k}$ can be represented as a matrix:

$\boldsymbol{k}=\left( \begin{matrix} k_{x} \\ k_{y} \\ k_{z} \end{matrix} \right)$ (S13)

Resolving Eq. S11 into $\left\{ x, y, z \right\}$ components:

$\left( k_{x}E_{x}+k_{y}E_{y}+k_{z}E_{z} \right)k_{x}-\left( k_{x}^{2}+k_{y}^{2}+k_{z}^{2} \right)E_{x}+k_{0}^{2}\epsilon_{\parallel}E_{x}=0$ (S14)

$\left( k_{x}E_{x}+k_{y}E_{y}+k_{z}E_{z} \right)k_{y}-\left( k_{x}^{2}+k_{y}^{2}+k_{z}^{2} \right)E_{y}+k_{0}^{2}\epsilon_{\parallel}E_{y}=0$ (S15)

$\left( k_{x}E_{x}+k_{y}E_{y}+k_{z}E_{z} \right)k_{z}-\left( k_{x}^{2}+k_{y}^{2}+k_{z}^{2} \right)E_{z}+k_{0}^{2}\epsilon_{\perp}E_{z}=0$ (S16)

By solving the above equations, dispersion relations for the ordinary waves ($E_{y}$, polarized along y)

$\frac{k_{x}^{2}+k_{y}^{2}+k_{z}^{2}}{\epsilon_{\parallel}}=k_{0}^{2}$ (S17)

and the extra-ordinary waves ($\boldsymbol{E}$ in the *xz* plane)

$\frac{k_{x}^{2}+k_{y}^{2}}{\epsilon_{\perp}}+\frac{k_{z}^{2}}{\epsilon_{\parallel}}=k_{0}^{2}$ (S18)

can be obtained^3,4^. For the TM polarized case (extra-ordinary waves), continuity of the tangential field components ($H_{y}$ and $E_{x}$) for the incident ($i$), reflected ($r$), and transmitted ($t$) waves between two anisotropic medium gives (${\bar{\boldsymbol{\epsilon}}}_{1}$and ${\bar{\boldsymbol{\epsilon}}}_{2}$):

$H_{i}\boldsymbol{+}H_{r}\boldsymbol{=}H_{t}$ (S19)

$E_{i}\boldsymbol{+}E_{r}\boldsymbol{=}E_{t}$ (S20)

From Eq. S10:

$E_{i}\boldsymbol{=}\frac{{k_{1z}H}_{y}}{\omega\epsilon_{0}\epsilon_{1\parallel}}$ (S21)

$E_{r}\boldsymbol{=}\boldsymbol{-}\frac{{k_{1z}H}_{y}}{\omega\epsilon_{0}\epsilon_{1\parallel}}$ (S22)

$E_{t}\boldsymbol{=}\frac{{k_{2z}H}_{y}}{\omega\epsilon_{0}\epsilon_{2\parallel}}$ (S23)

where $k_{iz}=\pm\sqrt{\epsilon_{i\parallel}k_{0}^{2}-\epsilon_{i\parallel}/\epsilon_{i\perp}k_{x}^{2}}$ ($i=$1, 2). Simplifying the above equations gives the reflection ($r_{a}$) and transmission ($t_{a}$) coefficients for an interface between ${\bar{\boldsymbol{\epsilon}}}_{1}$and ${\bar{\boldsymbol{\epsilon}}}_{2}$.

$r_{a}=\frac{\epsilon_{2\parallel}k_{1z}-\epsilon_{1\parallel}k_{2z}}{\epsilon_{2\parallel}k_{1z}+\epsilon_{1\parallel}k_{2z}}$ (S24)

$t_{a}=\frac{2\epsilon_{2\parallel}k_{1z}}{\epsilon_{2\parallel}k_{1z}+\epsilon_{1\parallel}k_{2z}}$ (S25)

**S2. Power dissipation-Dipole model**

*1. Power dissipation in an isotropic medium*

The power dissipation (energy loss, $dW/dt$) associated with inelastic tunneling can be obtained from the current density $\boldsymbol{J}$ and the electric field $\boldsymbol{E}$ as:

$\frac{dW}{dt}=-\frac{1}{2}\int_{v}\mathfrak{R}\left\{ \boldsymbol{J}^{\boldsymbol{*}}\boldsymbol{\cdot E} \right\}d^{3}r$ (S26)

where $\mathfrak{R}$ represents the real part. In the time harmonic response ($e^{i\omega t}$), for a dipole located at $r_{0}$, $\boldsymbol{J}\left( \boldsymbol{r} \right)\boldsymbol{=}\boldsymbol{-}i\omega\boldsymbol{\mu}\delta\left( \boldsymbol{r}-\boldsymbol{r}_{0} \right)$, where $\boldsymbol{\mu}$ represents the dipole amplitude. Writing $\boldsymbol{\mu}^{\boldsymbol{*}}\boldsymbol{=}\boldsymbol{\mu}_{real}\boldsymbol{-}{i\boldsymbol{\mu}}_{imag}$, it can be shown that

$\frac{dW}{dt}=\frac{\omega}{2}\mathfrak{I}\left\{ \boldsymbol{\mu}^{\boldsymbol{*}}\boldsymbol{\cdot E}\left( \boldsymbol{r}_{0} \right) \right\}$ (S27)

where $\mathfrak{I}$ represents the imaginary part. For a dipole located at $\boldsymbol{r}_{0}$, the electric field at $\boldsymbol{r}$, $\boldsymbol{E}\left( \boldsymbol{r} \right)$, can be expressed in terms of the dyadic Green’s function $\boldsymbol{G}$ as^5^

$\boldsymbol{E}\left( \boldsymbol{r} \right)=\omega^{2}\mu\mu_{0}\boldsymbol{G}\left( \boldsymbol{r},\boldsymbol{r}_{0} \right)\boldsymbol{\mu}$ (S28)

So, the power dissipation becomes:

$\frac{dW}{dt}=\frac{\omega^{3}}{2}\left| \boldsymbol{\mu} \right|^{2}\mathfrak{I}\left\{ \boldsymbol{n}_{\mu}\cdot\boldsymbol{G}\left( \boldsymbol{r},\boldsymbol{r}_{0} \right)\boldsymbol{n}_{\mu} \right\}$ (S29)

where $\boldsymbol{n}_{\mu}$represents the unit vector in the direction of $\boldsymbol{\mu}$. For the dipole source, the magnetic vector potential $\boldsymbol{A}\left( \boldsymbol{r} \right)$can be represented in terms of $\boldsymbol{G}$ as

$\boldsymbol{A}\left( \boldsymbol{r} \right)=-\mu_{0}i\omega\boldsymbol{\mu}\int\delta\left( \boldsymbol{r}^{\boldsymbol{'}}\boldsymbol{-}\boldsymbol{r}_{0} \right)\boldsymbol{G}\left( \boldsymbol{r}, \boldsymbol{r}^{\boldsymbol{'}} \right)dV^{\boldsymbol{'}}$ (S30)

$\boldsymbol{A}\left( \boldsymbol{r} \right)=-\mu_{0}i\omega\boldsymbol{\mu}\frac{e^{ik\left| \boldsymbol{r-}\boldsymbol{r}_{0} \right|}}{4\pi\left| \boldsymbol{r-}\boldsymbol{r}_{0} \right|}$ (S31)

From the angular representation of $\boldsymbol{A}\left( \boldsymbol{r} \right)$^6^:

$\boldsymbol{A}\left( \boldsymbol{r} \right)=-\frac{\mu_{0}\omega k_{0}}{8\pi^{2}}\boldsymbol{\mu}\iint\frac{e^{i\left( k_{x}\left( x-x_{0} \right)+k_{y}\left( y-y_{0} \right)+k_{z}\left| z-z_{0} \right| \right)}}{k_{z}}{dk}_{x}{dk}_{y}$ (S32)

Representing $\frac{1}{k_{0}}\left( k_{x}, k_{y}, k_{z} \right)$ as ($s_{x}, s_{y}, s_{z}$):

$\boldsymbol{A}\left( \boldsymbol{r} \right)=-\frac{\mu_{0}\omega k_{0}}{8\pi^{2}}\boldsymbol{\mu}\iint\frac{e^{ik_{0}\left( s_{x}\left( x-x_{0} \right)+s_{y}\left( y-y_{0} \right)+s_{z}\left| z-z_{0} \right| \right)}}{S_{z}}ds_{x}ds_{y}$ (S33)

From $i\omega\left( 1+\frac{1}{k_{0}^{2}}\boldsymbol{\nabla\nabla\cdot} \right)\boldsymbol{A}\left( \boldsymbol{r} \right)$, $\boldsymbol{E}\left( \boldsymbol{r} \right)$ can be obtained and comparing it with Eq. S28 will give the form of $\boldsymbol{G}\left( \boldsymbol{r},\boldsymbol{r}_{0} \right)$ in angular representation^5^,

$\boldsymbol{G}\left( \boldsymbol{r},\boldsymbol{r}_{0} \right)=-\frac{i}{8\pi^{2}}\iint\boldsymbol{M}e^{e^{ik_{0}\left( s_{x}\left( x-x_{0} \right)+s_{y}\left( y-y_{0} \right)+s_{z}\left| z-z_{0} \right| \right)}}ds_{x}ds_{y}$ (S34)

where $\boldsymbol{M}$represents the matrix for the angular spectrum components^5^.

$\boldsymbol{M}=\frac{1}{k_{z}}\left( \begin{matrix} 1-s_{x}^{2} & -s_{x}s_{y} & {\mp s}_{x}s_{z} \\ -s_{x}s_{y} & 1-s_{y}^{2} & {\mp s}_{y}s_{z} \\ {\mp s}_{x}s_{z} & {\mp s}_{y}s_{z} & 1-s_{z}^{2} \end{matrix} \right)$ (S35)

where $\mp$selection depends on the absolute value of $\left| z-z_{0} \right|$. Negative sign applies for $z-z_{0}>0$and a positive sign for $z-z_{0}<0$. For a $\hat{z}$ oriented dipole Eq. S34 can be simplified to:

$\boldsymbol{G}\left( \boldsymbol{r},\boldsymbol{r}_{0} \right)=-\frac{ik_{0}}{4\pi}\int_{0}^{1} ds\frac{s^{3}}{\sqrt{1-s^{2}}}$ (S36)

where $k_{0}=\frac{\omega}{c}$ and $s^{2}=S_{x}^{2}+S_{y}^{2}$. Evaluating the integral gives:

$\mathfrak{I}\left[ \boldsymbol{G}\left( \boldsymbol{r},\boldsymbol{r}_{0} \right) \right]=\frac{k_{0}}{6\pi}I$ (S37)

where $I$ represents the unity matrix. Thus $dW/dt$ becomes:

$\frac{dW}{dt}=\frac{\left| \boldsymbol{\mu} \right|^{2}\omega^{4}}{12\pi\epsilon_{0}c^{3}}$ (S38)

*2. Power dissipation in a uniaxial medium*

For the uniaxial medium, we follow Clemmow’s approach^2^ to obtain the vector potential in angular representation. Fields of a dipole in a uniaxial medium can be calculated from the corresponding fields in an isotropic medium by a proper scaling. Since the resultant field of a $\hat{z}$ oriented dipole in a tunnel junction is TM ($H_{z}=0$) in nature, we adopt Clemmow’s scaling approach, as used by Chance *et al*.^7^ for investigating dipole emitters embedded in uniaxial media and by Wasey *et al*.^8^ for investigating the effect of birefringence on the optical emission from thin-films. For the uniaxial media characterized by Eq. S12, angular representation of the vector potential is given by:

$\boldsymbol{A}\left( \boldsymbol{r} \right)=\frac{\mu_{0}\omega k_{0}}{8\pi^{2}}\boldsymbol{\mu}_{z}\frac{\epsilon_{\parallel}}{\epsilon_{\perp}}\iint\frac{e^{ik_{0}\left( s_{x}\left( x-x_{0} \right)+s_{y}\left( y-y_{0} \right)+s_{z}\left| z-z_{0} \right| \right)}}{S_{z}}ds_{x}ds_{y}$ (S39)

where the dispersion follows Eq. S18 and in terms of $s_{i}$

$\frac{S_{x}^{2}+S_{y}^{2}}{\epsilon_{\perp}}+\frac{S_{z}^{2}}{\epsilon_{\parallel}}=1$ (S40)

Following the same procedure for Green’s function evaluation as in Eq. S36, the decay rate $\gamma_{0}^{'}$($=\frac{1}{\hbar\omega}\frac{dW}{dt}$) of a dipole in the uniaxial medium is given by:

$\gamma_{0}^{'}=\frac{\left| \boldsymbol{\mu} \right|^{2}k_{0}^{3}}{12\pi\epsilon_{0}}\sqrt{\epsilon_{\parallel}}$ (S41)

*3. Au//hBN//Gr junction*

In the case of air-Au//hBN//Gr-glass system ($\epsilon_{0}-\epsilon_{Au}\left( \omega\right)-{\bar{\boldsymbol{\epsilon}}}_{hBN}-\epsilon_{Gr}-\epsilon_{Glass}$), for a dipole located in the uniaxial hBN layer, we calculate the normalized decay rate $\Gamma^{'}$($={\gamma^{'}/\gamma}_{0}^{'}$) from $\boldsymbol{G}^{\boldsymbol{'}}\left( \boldsymbol{r}_{0},\boldsymbol{r}_{0} \right)$ (Eq. S34) obtained from the reflected fields of the individual plane waves in the angular spectrum. Normalized decay rate $\Gamma^{'}$ for a $\hat{z}$ oriented dipole ($\mu^{\perp}$) can be obtained as (for a quantum efficiency of $\eta_{0}$)^9^:

$\Gamma^{'}=1-\eta_{0}+\eta_{0}\frac{3}{2}\frac{\sqrt{\epsilon_{\parallel}}}{\epsilon_{\perp}^{2}}Re\int_{0}^{\infty} ds\frac{s}{s_{z3}}\left[ \frac{\mu_{\perp}^{2}s^{2}\left[ 1+R_{345}^{p}exp\left( -2ik_{0}s_{z3}Z_{3} \right) \right]\left[ 1+R_{321}^{p}exp\left( 2ik_{0}s_{z3}Z_{2} \right) \right]}{1-{R_{345}^{p}R}_{321}^{p}exp\left( 2ik_{0}s_{z3}L_{3} \right)} \right]$ (S42)

where $s_{zi}=\sqrt{\epsilon_{i}-s^{2}}$. A five-layer stratified configuration is used in the calculation to represent the air(1)-Au(2)-hBN(3)-Gr(4)-glass(5) system with the corresponding indices 1-5 used to represent the dielectric functions. Interpolated data set from Johnson and Christy^10^ is used for the Au dielectric function. A dielectric medium with refractive index values of 1.5 is used to represent the glass-substrate and $R_{ijk}^{p}$represents the effective reflection coefficient for the top/bottom 3-layer medium formed by $\varepsilon_{i}$, $\varepsilon_{j}$, and $\varepsilon_{k}$ ($ijk=$321 or 345) with respect to the dipole’s location^5^:

$R_{ijk}^{p}=\frac{r_{ij}^{p}+r_{jk}^{p}\exp\left( 2ik_{0}s_{iz}d \right)}{1+r_{ij}^{p}r_{jk}^{p}\exp\left( 2ik_{0}s_{iz}d \right)}$ (S43)

where $d$ represents the thickness of $j^{th}$ layer and $r_{ij}^{p}$ and $r_{jk}^{p}$ represents the single interface reflection coefficients as given by Eq. S24. The thickness of the hBN layer is represented as $L_{3}$ and $z_{2}=z_{3}=L_{3}/2$ represents the dipole location in hBN with respect to the Au or Gr electrodes.

**S3. Radiative decay rate**

From the time averaged Poynting vector

$\left\langle\boldsymbol{S}\left( t \right) \right\rangle=\frac{1}{2}\left\{ \boldsymbol{E}\times\boldsymbol{H}^{\boldsymbol{*}} \right\}$ (S44)

radiative decay rate $\Gamma_{R}^{'}$($=\frac{1}{\gamma_{0}^{'}}\int ds\left\langle\boldsymbol{S}\left( t \right) \right\rangle$) for the anisotropic case is given by:

$\Gamma_{R}^{'}=\eta_{0}\frac{3}{4}\frac{\epsilon_{\parallel}^{3/2}}{\epsilon_{5}\epsilon_{\perp}^{2}}\int_{0}^{\sqrt{\epsilon_{\perp}}} s^{3}s_{z5}\left| \frac{S_{\uparrow}}{s_{z3}}\exp\left( ik_{0}s_{z3}L_{3} \right) \right|^{2}ds$ (S45)

where

$S_{\uparrow}=t_{35}\frac{\left( 1-r_{32}e^{ik_{0}s_{z3}L_{3}} \right)}{\left( 1-r_{32}r_{35}e^{2ik_{0}s_{z3}L_{3}} \right)}$ (S46)

where the effect of the Gr layer is neglected in calculating the radiated power into the glass substrate.

**S4. Multilayer Graphene: Optical properties**

From the review of Emani *et al*.^11^, multilayer graphene offers improved tunability in optical response and conductivity. The authors also note that studies on interlayer coupling and screening in exfoliated multilayer graphene follows the linear band structure model up to four layers and preserve the unique optical and electrical properties of graphene for few layers^12,13^. To treat the multilayer graphene as an effective medium, the conductivity $\sigma\left( \omega\right)$ can be used to represent the effective permittivity as^14^

$\epsilon\left( \varepsilon\right)=1+i\frac{\sigma\left( \omega\right)}{\omega\epsilon_{0}t_{G}}$ (S49)

where $t_{G}$ represents the thickness of the multilayer graphene (for a three-layer system $t_{G}$ ≈ 3×0.34 nm ≈ 1 nm). Following the method given by Hanson^15^ and Vakil and Engheta^14^ for multilayer graphene conductivity, and assuming that the multilayer graphene conductivity is independent of the photon energy > 1 eV^12^, we can write

$\sigma\approx e^{2}/4\hbar$ (S50)

and

$\epsilon\left( \varepsilon\right)=1+i\frac{e^{2}/4\hbar}{\omega\epsilon_{0}t_{G}}$ (S51)

which accounts for the 2.3% absorption in the graphene layer. To implement the graphene conductivity in a numerical simulation, we follow the method proposed by Emani *et al*.^11^.

**S5. Sample fabrication**

The Au//hBN//Gr tunneling junction device was fabricated on a commercial glass coverslip (Marienfield, 160 μm thick), according to the procedure illustrated in Fig. S1. Few-layer graphene and hBN flakes were mechanically exfoliated on a polymethylglutarimide/polymethyl-methacrylate (PMGI/PMMA) sacrificial double-layer, from commercial natural graphite (NGS Naturagraphit GmBH) and hBN (HQ Graphene) crystals. A graphene flake with thicknesses of ~1.0 nm (corresponding to 3 layers) was chosen as the bottom electrode. The flake was lifted on the PMMA layer by dissolving the PMGI layer with MF319. Then it was transferred to the glass coverslip, afterward, the PMMA film was removed by acetone (Fig. S1a). The graphene flake was patterned to a 5 μm wide strip using O_2_ plasma (Femto Science, VITA) etching with a PMMA mask (Fig. S1b-c). The mask was fabricated by electron beam lithography (EBL, JEOL, JBX-6300FS), with bilayer PMMA resist (PMMA 495 A3 and PMMA 950 A5, prebake at 180˚C for 5 min and 2 min, respectively), followed by resist development in MIBK:IPA (1:3) for 30 s and IPA for 30 seconds. Afterward, an hBN flake with thicknesses of ~2.3 nm (corresponding to 7 layers) was transferred onto the top of the graphene electrode as the tunneling barrier (Fig. S1d). The transferred hBN//Gr assembly was then annealed in a vacuum at 250 °C for 12h. The 5 μm wide top electrode was patterned again by EBL (Fig. S1e). After resist development, the Au layer (60 nm thick) with a Ti adhesion layer (1 nm thick) was deposited using thermal evaporation (Kurt J. Lesker, NANO 36) followed by lift-off in acetone (Fig. S1f). Finally, a 5 μm ×5 μm junction area was defined by the overlapping area of graphene and Au strips.


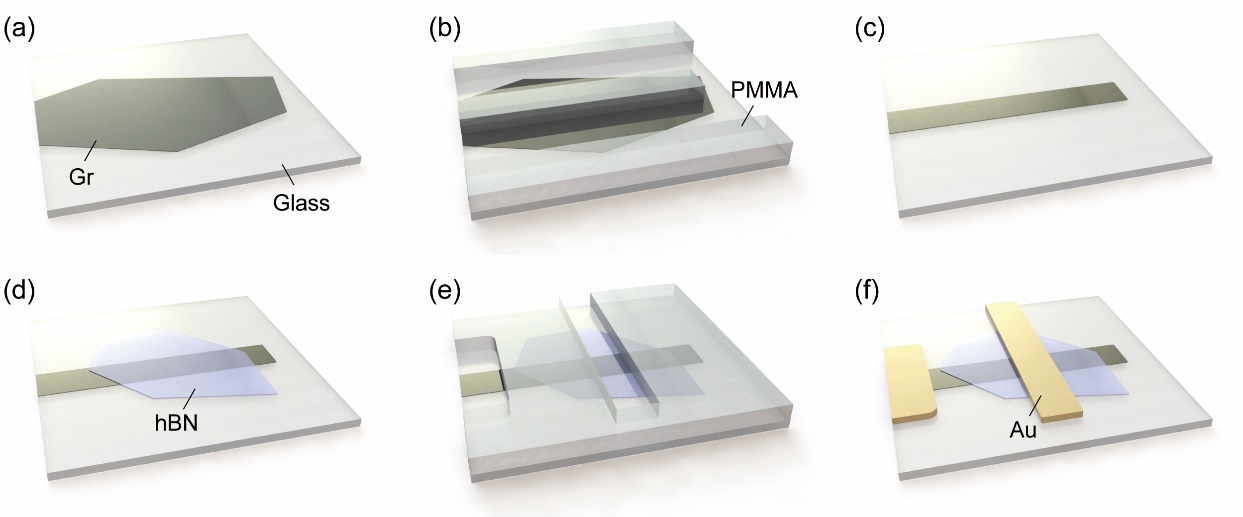


**Figure S1.** Fabrication procedures of the Au//hBN//Gr tunneling junction device. A few-layer graphene flake was transferred to a glass substrate **(a)** and then etched to be a strip with a PMMA mask made by EBL **(b, c)**. The hBN flake was then placed on the graphene strip **(d)**. Finally, the Au electrode was fabricated on the top of the stack **(e, f)**.

Fig. S2 shows the atomic force microscope (AFM) images of the fabricated device. The AFM image of the junction area shows where Au electrode overlaps with the hBN//Gr layers. The AFM shown in Fig. S2b represents the topography of the truncated end of the 5 $\mu$m wide Au waveguide. The height profile of the Au waveguide along the red line shown in Fig S2b is plotted as the inset and the estimated thickness is ~ 65 nm. Topography of the hBN flake is shown in Fig. S2c and the estimated height from the height profile given in the inset is ~ 2.3 nm.

**
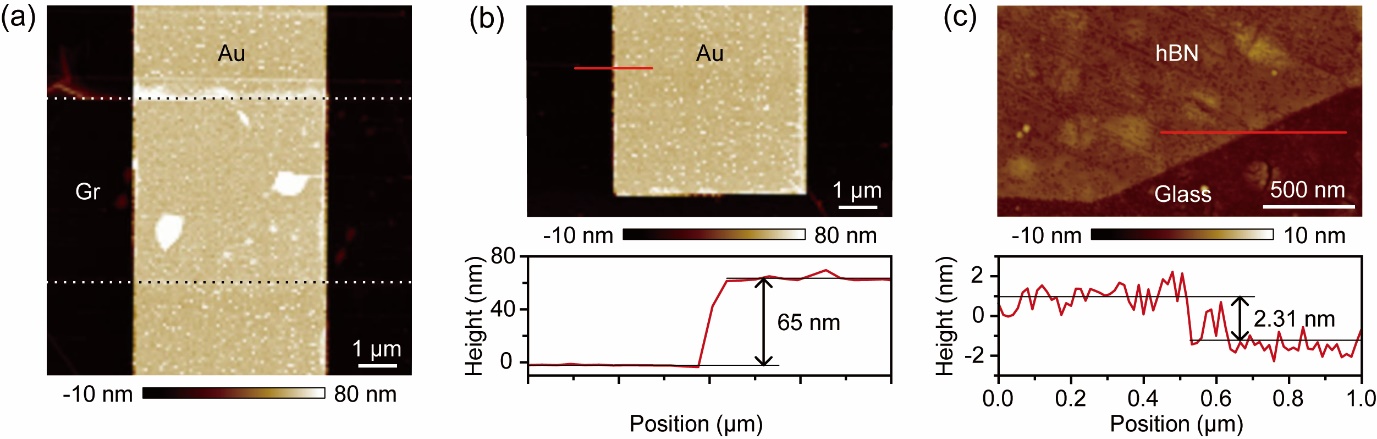
**

**Figure S2.** AFM images of **(a)** the junction area, **(b)** the Au waveguide of the Au//hBN//Gr tunneling junction device, and (**c**) the topography of the hBN flake. Insets to (**b**) and (**c**) show height profile along the red lines. The estimated thickness of the Au is ~ 65 nm and of hBN is ~ 2.3 nm.

**S6. Electrical and optical characterization**

The time average of the current-time traces *I*(t) recorded over 120 s for ${\pm V}_{b}$ in the range $\pm$1.5 to $\pm$2.0 V is shown in Fig. S3. The noise level in the time-average response over 120 s is minimal and we attribute this to the stable tunnel contact formed between hBN and Gr^16^. We annealed the hBN-Gr to stabilize the device performance, which is the same as the post-transfer treatment reported in the past^17,18^. One difference is we transferred the hBN-Gr assembly followed by deposition of the Au electrodes instead of transferring the van der Waals heterostructure on the Au. In principle, this change improves the contact between the Au and van der Waals heterostructure and likely improves the electrical stability.

The devices were optically characterized using an inverted microscope (Nikon, Eclipse Ti-E) equipped with an EMCCD (Andor, iXon Ultra 897) and a spectrometer (Andor, Shamrock 303i). The wavelength dependent detection efficiency (η_op_) of the optical system for the spectra collection is shown in Fig. S4. The device emissions were collected from the backside of the substrates with an oil-immersed objective (Nikon, 100×, NA 1.49), when the devices were biased using a source meter (Keithley 6430). Real- and Fourier-plane images were captured by projecting the corresponded image plane and the back focal plane of the objective on the EMCCD. Real and BFP images of the light emission corresponding to bias voltages in the range of ±1.5 to ±1.8 V are shown in Fig. S5.


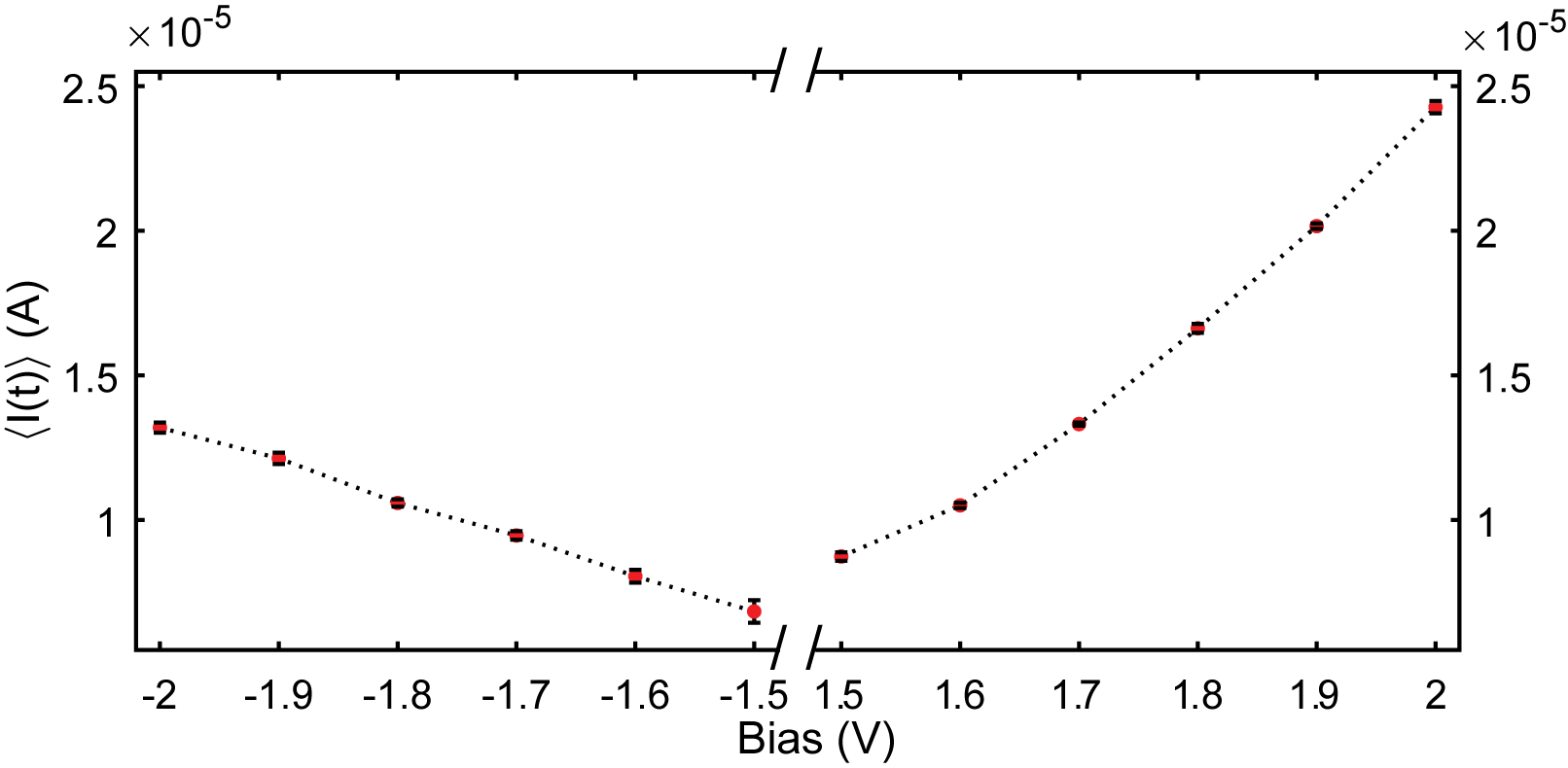


**Figure S3.** The time average of the tunnel current over 120 s for both $+V_{b}$ and $-V_{b}$. Error bars represent the standard deviation.


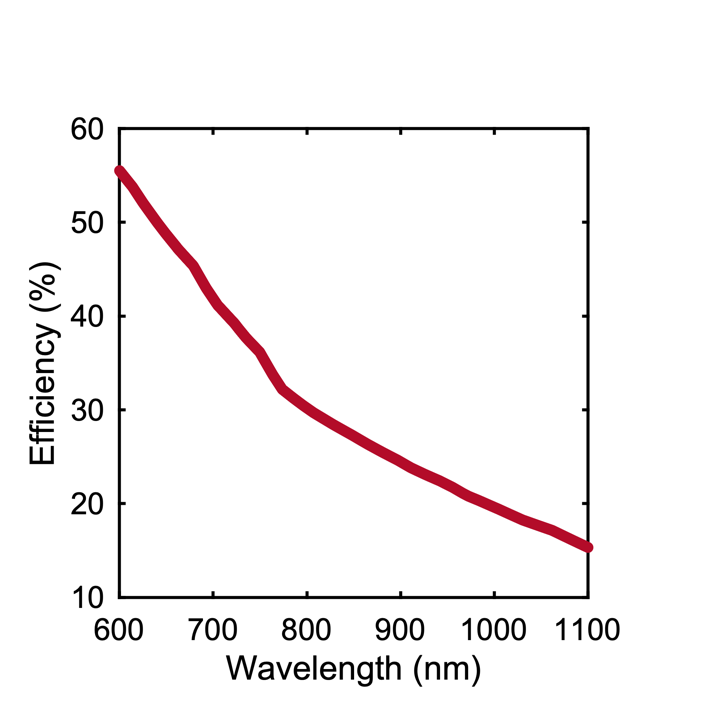


**Figure S4.** Wavelength dependent detection efficiency of the optical system used for collecting the spectra (shown in Fig. 4c).


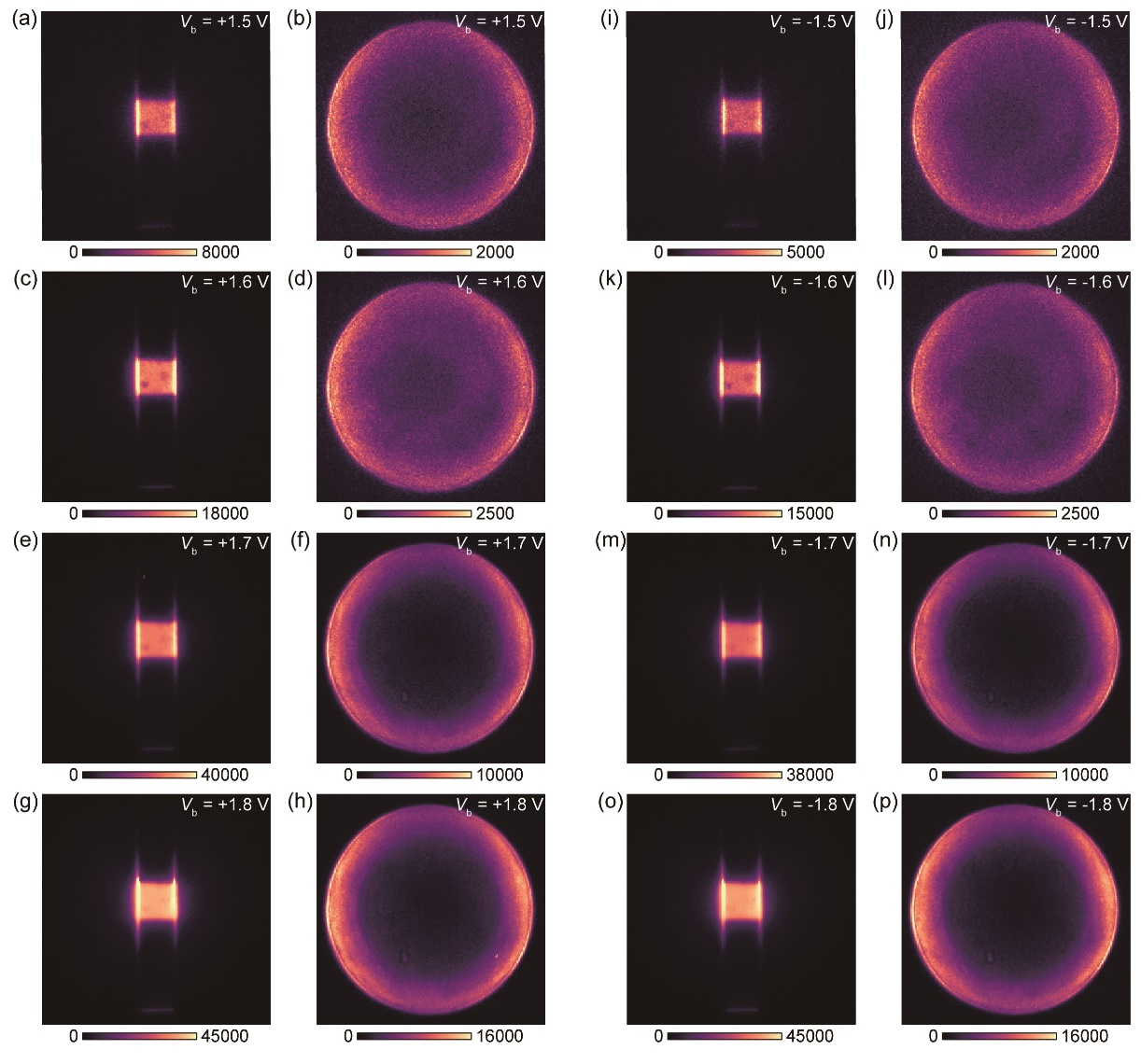


**Figure S5. (a-p)** Real-plane and the corresponding Fourier-plane images of the emission recorded with the EMCCD for bias voltages in the range of ±1.5 V to ±1.8 V. Intensity scales are adjusted give best possible contrast for the emission features.

**S7. Emission spectra: Quantum cutoff**

To determine the emission cutoff for the experimental spectra (Fig. 4c), we fitted the decaying tail (at the high energy side, below 850 nm) of each spectrum to a Gaussian distribution. Figure S5 shows the spectra (open circles) and the solid lines represent the partial Gaussian fit. To demarcate the baseline for the emission cutoff, average emission intensity above the theoretical cutoff ($\hbar\omega=|eV_{b}|$) is evaluated from each data set, corresponding to each $V_{b}$. The cumulative mean of these intensities is defined as the noise floor of the emission spectra (horizontal line in Fig. S5). The point at which the partial Gaussian fits are intersecting the noise floor is defined as the experimental quantum cutoff for the corresponding spectra.

**
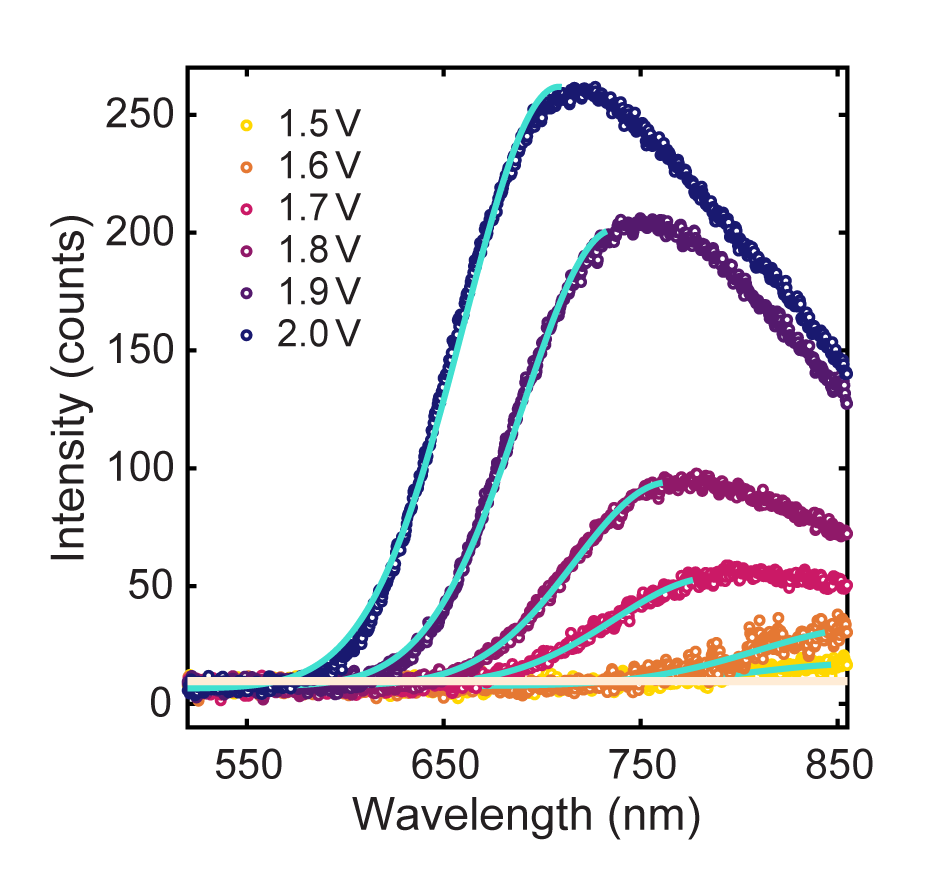
**

**Figure S6.** Emission spectra for $+V_{b}$ (open circles) and the partial Gaussian fit (solid lines) below 850 nm. The horizontal line represents the calculated baseline, representing the noise floor.

**S8. Spectral efficiency and Radiative power- Experiment**

1. *Spectral efficiency - Experiment*

The spectral efficiency is defined as the number of photons detected per tunneling electrons and is obtained from the emission intensity shown in Fig. 4c of the main text. The spectra are corrected for the detection efficiency (η_op_) of the optical system (Fig. S4). Average photon counts per pixel for the EMCCD/CCD is calculated from the CCD counts ($\eta_{i}$) using the following method^19^. For the EMCCD images, the number of photons per seconds per pixel is given by

$\chi_{i}=\frac{{(\eta}_{i}-\mathrm{offset})\times\mathrm{sensitivity}}{QE\times EM Gain\times int. time}$ (S52)

where the offset represents the bias offset for the EMCCD defining the baseline for the camera and is obtained from a dark image, the $\mathrm{sensitivity}$ is defined in terms of the electrons/counts and is specific to the preamp settings of the EMCCD readout, the $\mathrm{QE}$ represents the quantum efficiency of the detector, the $EM Gain$ is the electron multiplying gain of the EMCCD and $int. time$ represents the integration time for EMCCD image acquisition. The length of the pixel (26 μm for the CCD of the spectrometer) is used to normalize $\chi_{i}$. The ratio of normalized $\chi_{i}$ to the number of electrons tunneling per second gives the spectral efficiency.

2. *Radiative power - Experiment*

The radiative power for the emission is obtained from the EMCCD images recorded in the BFP. From $\chi_{i}$ (Eq. S52), the radiative power $P_{i}$ per tunneling electron is obtained as:

$P_{i1}(1\leq s\leq1.49)=(1/n_{e})\int_{1}^{1.49} \int_{1}^{1.49} \chi_{i} ds_{x}ds_{y}$ (S53)

$P_{i2}(s\leq1)=(1/n_{e})\int_{0}^{1} \int_{0}^{1} \chi_{i} ds_{x}ds_{y}$ (S54)

where $P_{i1}$ represents the radiative power for $1\leq s\leq1.49$ and $P_{i2}$ is the radiative power within the critical angle ($s\leq1)$and $n_{e}$ represents the number of electrons tunneling per second. Since the theory calculates the normalized radiative power, for a meaningful comparison, we calculate the normalized radiative power from the experimental data, for which $P_{i1}$ and $P_{i2}$ are normalized with the radiative power of a dipole located in a homogeneous space$\left( P_{0}=\frac{\left| \boldsymbol{\mu} \right|^{2}\omega k_{0}^{3}}{12\pi\epsilon_{0}}\sqrt{\epsilon_{\parallel}} \right)$. The current dipole amplitude $|\boldsymbol{\mu|}$ (in units of $A.m$) is calculated from the time average tunneling current $\left\langle I\left( t \right) \right\rangle$ corresponding to each $\pm V_{b}$(Fig. 4b inset). To represent the tunnel current as an oscillating current element^4^, we use $\left| \boldsymbol{\mu} \right|\boldsymbol{=}\frac{\left\langle I\left( t \right) \right\rangle\timesⅆl}{2\times\omega}$, where $ⅆl$ represents the length of the current element which is approximately the thickness of the hBN layer (tunneling length-scale) and $\omega$ is the frequency of dipole oscillation.

3. *SPP coupling efficiency - Experiment*

From the real plane EMCCD images (Fig. 4d, 4g, and Fig. S5) emitted power from the tunnel junction area (${P_{i}}_{jn}$) and from the Au waveguide-end (${P_{i}}_{wg}$) are obtained as a function of applied bias. ${P_{i}}_{wg}$ is corrected for the SPP propagation losses associated with the Au waveguide ($L_{wg}$~ 12.5 μm) for SPP propagation length $\Lambda_{SPP}$ ~ 5.2 μm^20^. A factor of 4 is also included to account for the SPP power coupled to all in-plane directions from the tunnel junction area. The relative SPP coupling efficiency Π_SPP_ is defined as:

${}_{\mathrm{SPP}}=\frac{{{4\times P}_{i}}_{wg}\times\exp(L_{wg}/\Lambda_{SPP})}{{{4\times P}_{i}}_{wg}\times\exp(L_{wg}/\Lambda_{SPP})+{P_{i}}_{jn}}$ (S54)

**S9. Power spectral density and spectral efficiency - Theory**

1. *Power spectral density - Theory*

The rate of inelastic tunneling $(\gamma_{inel}$) is related to the power spectral density $P_{I}(\omega)$ of the current fluctuations as^21^ $\gamma_{inel}\propto P_{I}(\omega)$. Neglecting the spatial dependence of the electron wavefunction in parallel direction to the tunneling^22^, $P_{I}\left( \omega\right)$ for the emission can be represented in terms of the experimental *I(V)* from the fluctuation-dissipation theorem^23^. For the finite temperatures (T > 0)^24,25^:

$P_{I}\left( \omega\right)=\frac{I\left( eV_{b}-\hbar\omega\right)}{1-\exp\left[ \frac{eV_{b}}{k_{B}T}\left( 1-\frac{\hbar\omega}{eV_{b}} \right) \right]}$ (S55)

where $k_{B}$ is the Boltzmann constant. Experimental *I(V)* from Fig. 4b is fitted to the Simmons’ model^24,26^ as shown in Fig. S7a. Then from Eq. S55, $P_{I}\left( \omega\right)$ for $\pm V_{b}$ is calculated as shown in Fig. S7b. In terms of the partial LDOS ($\rho_{p}),$ $\gamma_{inel}$ can be represented as^27^

$\gamma_{inel}=\frac{\pi}{12\epsilon_{0}}\rho_{p}\times P_{I}(\omega)$ (S56)


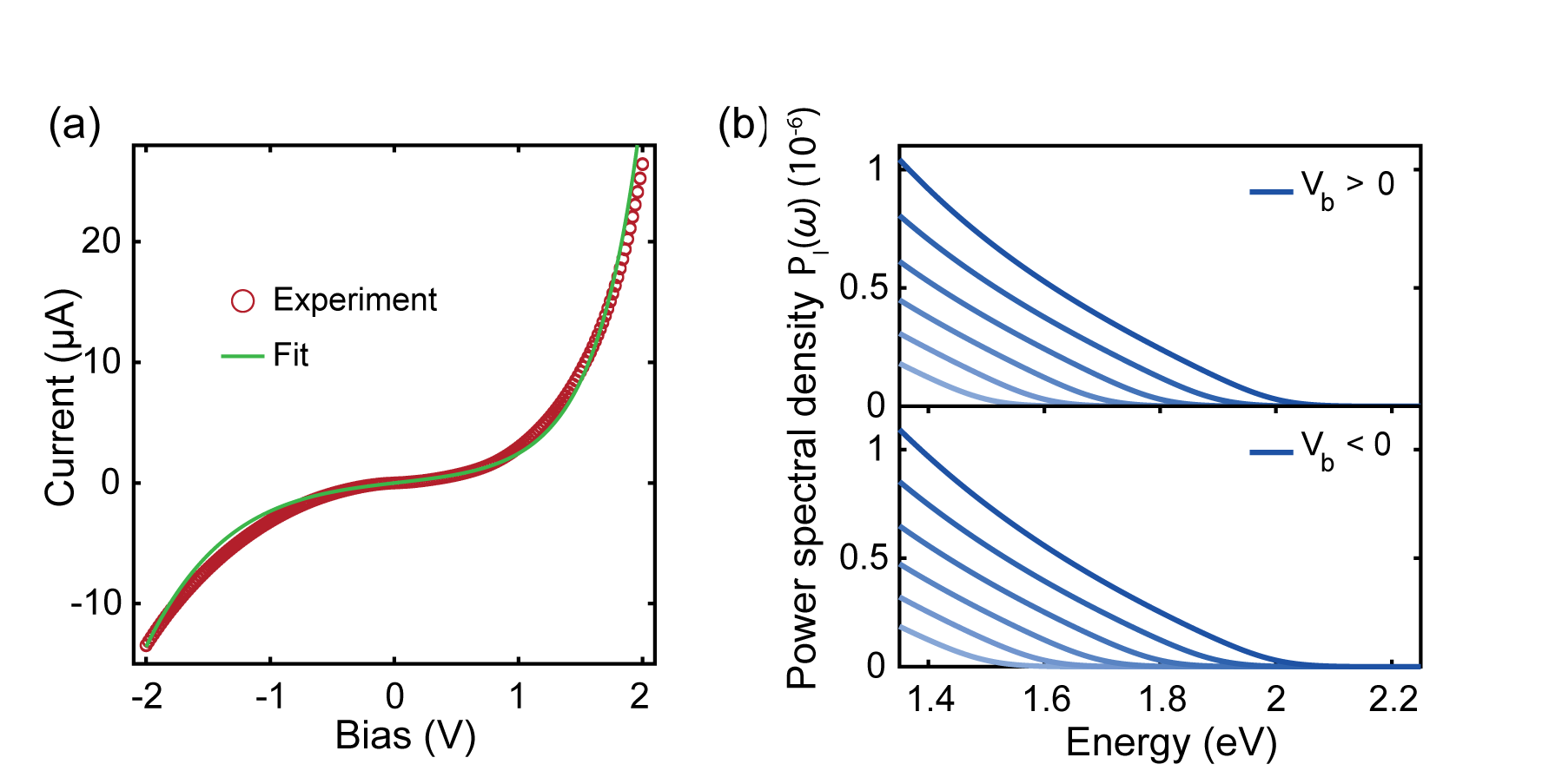


**Figure S7. (a)** Experimental I*(V)* data from Fig. 4a (open circles). Fitted curve (solid line) based on the Simmons’ model for a finite temperature (T = 300 K). Fitting parameters: for the barrier thickness of 2.3 nm (hBN thickness), mean barrier height of 2.49 eV and effective electron mass 0.49m_e_, where m_e_ is the free electron mass. **(b)** Power spectral density of the tunnel current fluctuations calculated from Eq. S57 for $\pm V_{b}$, from 1.5 V (light blue) to 2.0 V (dark blue) in steps of 0.1 V.

2. *Spectral efficiency and Radiative power - Theory*

From Eq. S56, the spectral efficiency from theory ($\Upsilon_{\mathrm{th}}$) can be represented in terms of the radiative decay rate $\Gamma_{R}^{'}$(anisotropic):

$\Upsilon_{\mathrm{th}}=\left( \frac{1}{\hbar\omega} \right)\frac{\pi}{12\epsilon_{0}}\rho_{0}\frac{P_{I}(\omega)}{\gamma_{el}}\Gamma_{R}^{'}$ (S57)

where $\gamma_{el}$ represents the elastic tunneling rate. For the isotropic case $\Gamma_{R}^{'}$ is replace by $\Gamma_{R}$. For comparing the spectral efficiency with the results from Parzefall *et al*.^18^,we calculate the spectral efficiency for Au//hBN//Gr TJ with glass replaced by air medium (n = 1). Figure S8 shows the calculated spectral efficiency (Eq. S57) for the positive (top panel) and negative applied bias (bottom panel). For calculation, $\Gamma_{R}^{'}$obtained for the Au//hBN//Gr TJ with air as the bounding medium on top and bottom is used, with $P_{I}\left( \omega\right)$ given by Eq. S55.

For the normalized radiative power shown in Fig. 5b and 5c, $\Gamma_{R}^{'}$(anisotropic) and $\Gamma_{R}$ (isotropic) are multiplied by the corresponding $P_{I}\left( \omega\right)$ (Eq. S55) to account for the inelastic tunneling efficiency and then integrated over the energy/wavelength.


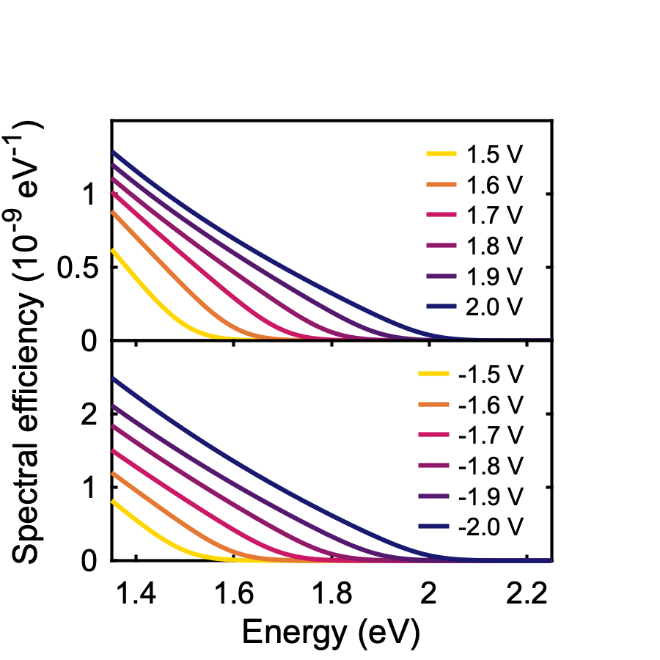


**Figure S8.** Spectral efficiency calculated for the Au//hBN//Gr system with glass replaced by air, for the positive (top panel) and negative applied bias (bottom panel).

3. *SPP coupling efficiency – Theory*

For SPP coupling efficiency calculation, we follow Eq. S57, from which spectral efficiency for the SPP emission ($\Upsilon_{\mathrm{spp}}$) is obtained by replacing $\Gamma_{R}^{'}$ by $\Gamma_{SPP}^{'}$:

$\Upsilon_{\mathrm{spp}}=\left( \frac{1}{\hbar\omega} \right)\frac{\pi}{12\epsilon_{0}}\rho_{0}\frac{P_{I}(\omega)}{\gamma_{el}}\Gamma_{SPP}^{'}$ (S58)

The relative SPP coupling efficiency Π_SPP_Th_ is defined as:

${}_{SPP\_Th} = \frac{\Upsilon_{\mathrm{spp}}}{{\Upsilon_{\mathrm{th}}+\Upsilon}_{\mathrm{spp}}}$ (S59)

**S10. Back focal plane: Intensity contributions**

The momentum space coordinates of the experimental BFP images (Fig. S9a) are calibrated with *k/k_0_* values obtained from the BFP image for the reflected light from an Au-glass interface^28^. Figure S9b shows the intensity line profiles evaluated along the dotted lines (1) and (2) shown in Fig. S9a. The line profile (2) represents the direct light emission contribution, with has a negligible contribution around s_y_ ~ -1.5 from the SPP scattering at the waveguide-end (See Fig. 4, main text). Line profile (1) includes both direct light emission from the junction area and SPP scattering contributions from the junction edges. From the line profile (1) it is evident that the emission beyond the critical angle along $\pm s_{x}$ has significant contributions from the SPP scattering from the junction edges and are localized at high angles (close to the NA=1.49 of the objective).

**
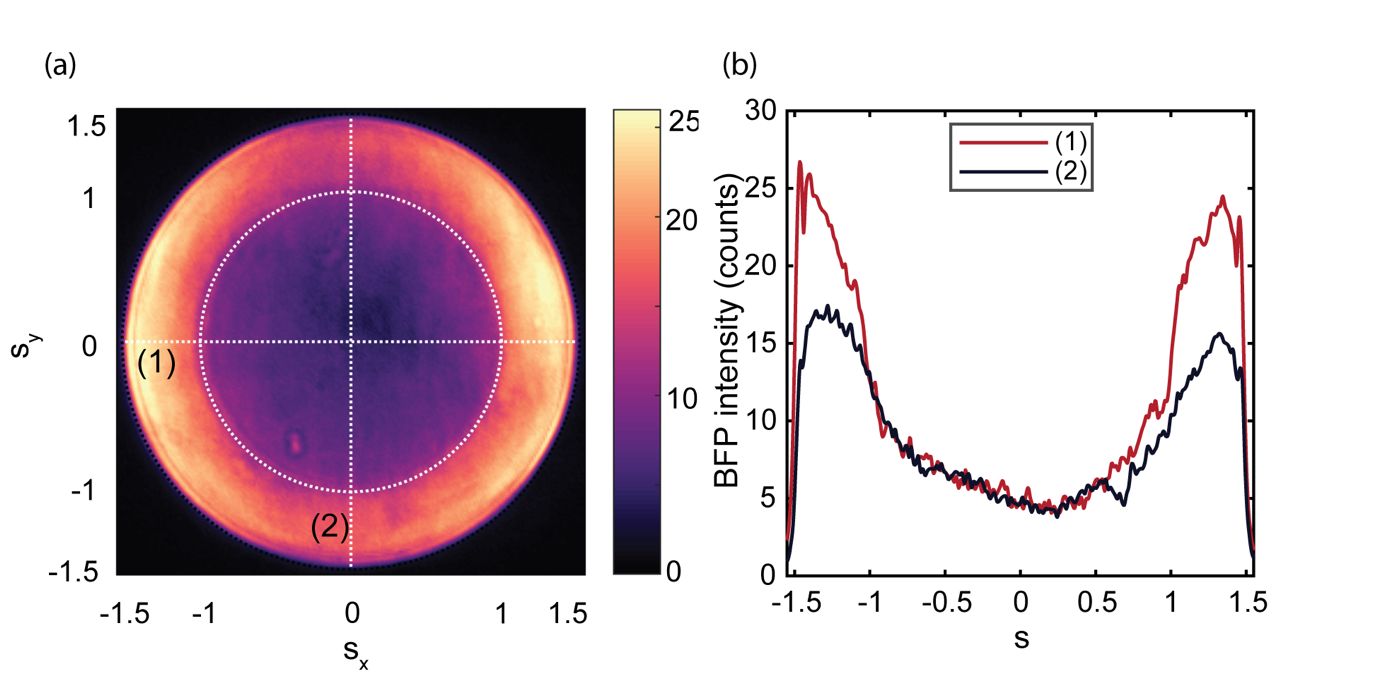
**

**Figure S9.** Intensity contributions in BFP (**a**) Calibrated BFP image for $V_{b}=2.0 V$. (**b**) Intensity evaluated along the dotted lines (1) and (2) shown in (**a**). Line profile (2) represents the direct light emission contribution, with negligible contribution around $s \sim-1.5$ from the SPP scattering at the waveguide-end (See Fig. 4, main text). Line profile (1) includes both direct light emission from the junction area and SPP scattering contributions from the junction edges.

**S11. Finite element modeling**

The wave optics module of the commercial software package COMSOL Multiphysics^29^ is used to calculate the radiative/non-radiative power with the finite element method (FEM) for the dipole embedded in isotropic/anisotropic medium. The simulation domain consists of a 4-layer system for the tunnel junction geometry (Au//hBN//Gr-glass) in 2D (***x****,* ***y***) plane (Fig. S10). The total simulation area is $2 \mu$m$\times2 \mu$m with a perfectly matched layer of thickness 500 nm defining the domain boundaries. Drude-Lorentz model is used to represent the Au electrode and Gr is represented as described in S4. Figure S10 shows the relative change in the intensity of the SPP field profile from the FEM modeling for the anisotropic (Fig. S10a) related to the isotropic case (Fig. S10b).

**
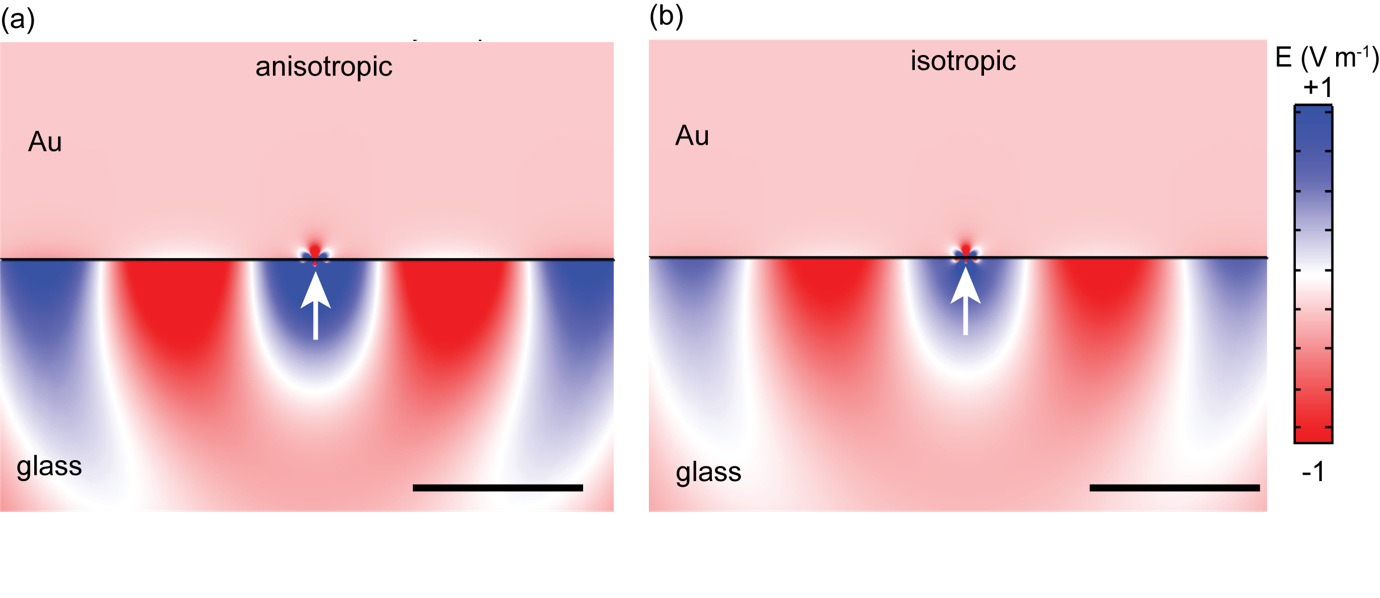
**

**Figure S10.** SPP field profile from the FEM simulation for the anisotropic (**a**) and isotropic case (**b**). Scale bar represents 200 nm and the vertical arrows denote the dipole’s positions. Only Au and glass domains are explicitly shown in the figure because of the small-scale thickness of the hBN and Gr domains.

**References**

1 Chew, W. C. *Waves and fields in inhomogeneous media*. (Wiley-IEEE Press, 1995).

2 Clemmow, P. C. *The plane wave spectrum representation of electromagnetic fields*. (Pergamon, 1966).

3 Born, M. & Wolf, E. *Principles of optics: Electromagnetic theory of propagation, interference and diffraction of light*. 7th edn, (Cambridge: Cambridge University Press, 1999).

4 Jackson, J. D. *Classical electrodynamics*. 3rd edn, (New York: John Wiley & Sons, 1999).

5 Novotny, L. & Hecht, B. *Principles of nano-optics*. 2nd edn, (Cambridge: Cambridge university press, 2012).

6 Mandel, L. & Wolf, E. *Optical coherence and quantum optics*. 1 edn, (Cambridge: Cambridge university press, 1995).

7 Chance, R. R., Prock, A. & Silbey, R. Molecular fluorescence and energy transfer near interfaces. in Advances in chemical physics, Volume 37 (eds Prigogine, I. & Rice, S. A.) (Weinheim: John Wiley & Sons, Inc., 1978), 1-65.

8 Wasey, J. A. E. *et al.* Effects of dipole orientation and birefringence on the optical emission from thin films. *Optics Communications* **183**, 109-121 (2000).

9 Ford, G. W. & Weber, W. H. Electromagnetic interactions of molecules with metal surfaces. *Physics Reports* **113**, 195-287 (1984).

10 Johnson, P. B. & Christy, R. W. Optical constants of the noble metals. *Physical Review B* **6**, 4370-4379 (1972).

11 Emani, N. K. *et al.* Graphene: A dynamic platform for electrical control of plasmonic resonance. *Nanophotonics* **4**, 214-223 (2015).

12 Mak, K. F. *et al.* The evolution of electronic structure in few-layer graphene revealed by optical spectroscopy. *Proceedings of the National Academy of Sciences* **107**, 14999-15004 (2010).

13 Sui, Y. & Appenzeller, J. Screening and interlayer coupling in multilayer graphene field-effect transistors. *Nano Letters* **9**, 2973-2977 (2009).

14 Vakil, A. & Engheta, N. Transformation optics using graphene. *Science* **332**, 1291-1294 (2011).

15 Hanson, G. W. Dyadic green’s functions and guided surface waves for a surface conductivity model of graphene. *Journal of Applied Physics* **103**, 064302 (2008).

16 Britnell, L. *et al.* Electron tunneling through ultrathin boron nitride crystalline barriers. *Nano Letters* **12**, 1707-1710 (2012).

17 Parzefall, M. *et al.* Antenna-coupled photon emission from hexagonal boron nitride tunnel junctions. *Nature Nanotechnology* **10**, 1058-1063 (2015).

18 Parzefall, M. *et al.* Light from van der waals quantum tunneling devices. *Nature Communications* **10**, 292 (2019).

19 *Andor support resources*, <https://andor.oxinst.com/assets/uploads/andor-support-resources/FAQ040.pdf>.

20 Du, W. *et al.* Highly efficient on-chip direct electronic–plasmonic transducers. *Nature Photonics* **11**, 623-627 (2017).

21 Parzefall, M. & Novotny, L. Optical antennas driven by quantum tunneling: A key issues review. *Reports on Progress in Physics* **82**, 112401 (2019).

22 Rendell, R. W. & Scalapino, D. J. Surface plasmons confined by microstructures on tunnel junctions. *Physical Review B* **24**, 3276-3294 (1981).

23 Rogovin, D. & Scalapino, D. J. Tunnel junction current fluctuations. *Physica* **55**, 399-404 (1971).

24 Kalathingal, V., Dawson, P. & Mitra, J. Scanning tunnelling microscope light emission: Finite temperature current noise and over cut-off emission. *Scientific Reports* **7**, 3530 (2017).

25 Zhang, C. *et al.* Antenna surface plasmon emission by inelastic tunneling. *Nature Communications* **10**, 4949 (2019).

26 Simmons, J. G. Generalized thermal j‐v characteristic for the electric tunnel effect. *Journal of Applied Physics* **35**, 2655-2658 (1964).

27 Bharadwaj, P., Deutsch, B. & Novotny, L. Optical antennas. *Advances in Optics and Photonics* **1**, 438-483 (2009).

28 Chen, H. Z. *et al.* Imaging the dark emission of spasers. *Science Advances* **3**, e1601962 (2017).

29 *Comsol inc.,*, Comsol multiphysics.
