# Supplementary material for: Optical Anisotropy in van der Waals materials: Impact on Direct Excitation of Plasmons and Photons by Quantum Tunneling
Source: Light Sci Appl. 2021 Nov 8;10:230. doi: 10.1038/s41377-021-00659-7 (PMC8575904; doi:10.1038/s41377-021-00659-7)

**Short summary**

Enhancement in local density of optical states arising from the optical anisotropy of hexagonal boron nitride significantly improves the emission rates of plasmons and photons in quantum mechanical tunnel junctions.

**Graphical abstract**


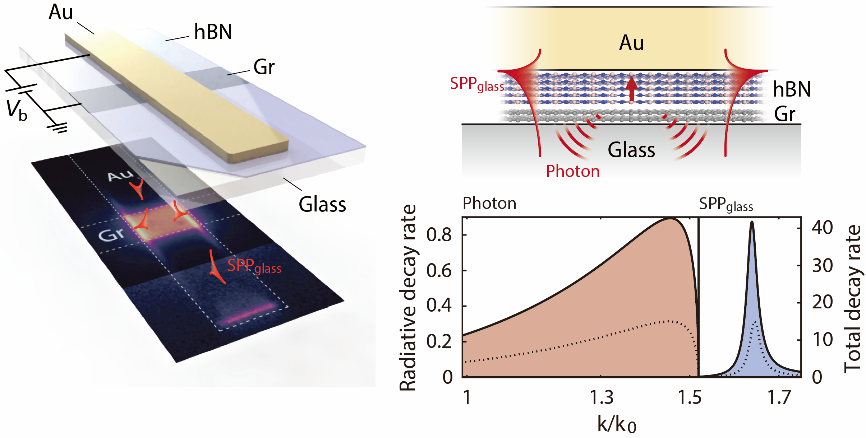

Supplement: Supplementary file 2 — Graphical abstract [file 41377_2021_659_MOESM2_ESM.docx]
